# Functional and dynamic profiling of transcript isoforms reveals essential roles of alternative splicing in interferon response

**DOI:** 10.1016/j.xgen.2024.100654

**Published:** 2024-09-16

**Authors:** Mahoko Takahashi Ueda, Jun Inamo, Fuyuki Miya, Mihoko Shimada, Kensuke Yamaguchi, Yuta Kochi

**Affiliations:** 1 Department of Genomic Function and Diversity, Medical Research Institute, Tokyo Medical and Dental University, Tokyo 113-8510, Japan; 2 Division of Rheumatology, University of Colorado School of Medicine, Aurora, CO, USA; 3 Department of Biomedical Informatics, Center for Health Artificial Intelligence, University of Colorado School of Medicine, Aurora, CO, USA; 4 Center for Medical Genetics, Keio University School of Medicine, Tokyo 160-8582, Japan; 5 National Center for Global Health and Medicine, Tokyo 162-8655, Japan; 6 Biomedical Engineering Research Innovation Center, Institute of Biomaterials and Bioengineering, Tokyo Medical and Dental University, Tokyo 113-8510, Japan; 7 Laboratory for Autoimmune Diseases, RIKEN Center for Integrative Medical Sciences, Yokohama, Kanagawa 230-0045, Japan; 8 Department of Allergy and Rheumatology, Graduate School of Medicine, The University of Tokyo, Tokyo, Japan

**Keywords:** long-read RNA-seq, isoform switching, interferon-stimulated genes, ISGs, alternative splicing regulation, PacBio Sequel II/IIe, intron retention

## Abstract

Type I interferon (IFN-I) plays an important role in the innate immune response through inducing IFN-I-stimulated genes (ISGs). However, how alternative splicing (AS) events, especially over time, affect their function remains poorly understood. We generated an annotation (113,843 transcripts) for IFN-I-stimulated human B cells called isoISG using high-accuracy long-read sequencing data from PacBio Sequel II/IIe. Transcript isoform profiling using isoISG revealed that isoform switching occurred in the early response to IFN-I so that ISGs would gain functional domains (e.g., *C4B*) or higher protein production (e.g., *IRF3*). Conversely, isoforms lacking functional domains increased during the late phase of IFN-I response, mainly due to intron retention events. This suggests that isoform switching both triggers and terminates IFN-I responses at the translation and protein levels. Furthermore, genetic variants influencing the isoform ratio of ISGs were associated with immunological and infectious diseases. AS has essential roles in regulating innate immune response and associated diseases.

## Highlights

• Long-read isoform annotation identifies >100,000 isoforms in IFN-I-stimulated B cells
• Alternative splicing alters isoform function at the translation and protein levels
• Isoform switching within ISGs triggers and terminates IFN responses over time
• Genetic variants affecting isoform ratio are linked to immune and infectious diseases

## Graphical abstract

**Figure:**
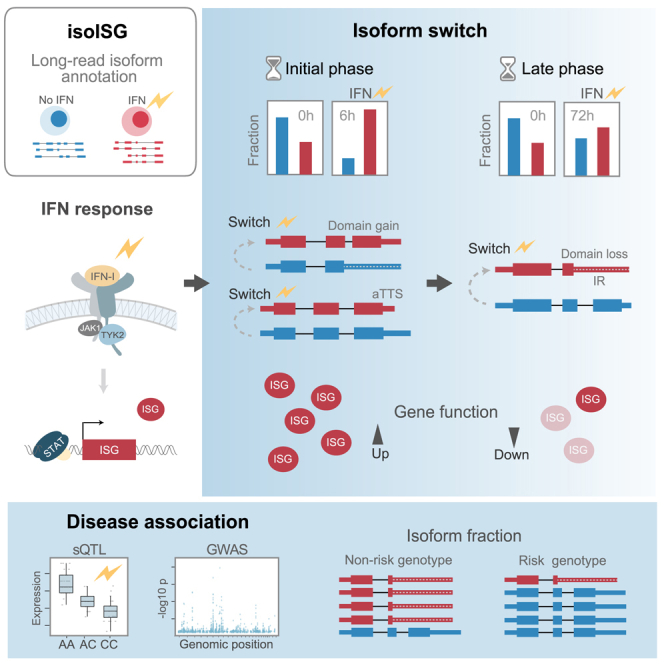

## Teaser

Ueda et al. discover that changes in transcript isoforms can alter the function of immune response genes in human B cells. These changes help explain how the body fights infections and regulates the immune system, emphasizing the role of gene splicing in health and disease.

## Introduction

Type I interferons (IFN-Is), such as interferon-α (IFN-α) and -β, are cytokines that trigger innate immune responses to protect against foreign pathogens as well as cancer cells, leading to the activation of the so-called IFN-stimulated genes (ISGs). IFN-I stimulation is mediated through the Janus kinase (JAK)/signal transducer and activator of transcription (STAT) pathway.^1^ STATs comprise a family of seven members (*STAT1–4*, *STAT5A/5B*, and *STAT6*) that function as dimeric transcription factors or in combination with other transcription factors. STATs form dimers (e.g., *STAT1*, *STAT2*), translocate into the nucleus, and induce the expression of a variety of ISGs, such as inflammatory cytokines, chemokines, and complement factors, via binding to IFN-stimulated response elements (ISREs).^2^^,^^3^^,^^4^ Although IFN-I is essential for the clearance of viruses, the dysregulation of IFN-I responses is known to underlie the pathogenesis associated with many autoimmune diseases such as rheumatoid arthritis (RA), systemic lupus erythematosus (SLE), Sjogren’s syndrome, and myositis.^5^^,^^6^^,^^7^ IFN-Is themselves are regulated by the IFN regulatory factor (IRF) gene family, such as *IRF3* and *IRF7*, upon stimulation of pattern-recognition receptors. Moreover, IFN-Is also increase the expression of *IRF7* in a well-documented forward feedback loop to enhance their own expression.^8^ Despite a number of studies showing the mechanism of ISG upregulation, less is known about the mechanism by which IFN responses are terminated, which may be critical to understanding the pathomechanism of IFN-I-mediated diseases.

Alternative splicing (AS), which produces multiple transcript isoforms (hereafter referred to as isoforms) from a single gene, has been recognized as a mechanism that can regulate the immune system.^9^^,^^10^^,^^11^^,^^12^ AS events are regulated in cell-specific and signal-specific manners.^13^^,^^14^ In regard to IFN responses, AS was characterized by changes in the alternative first exon in monocytes.^15^ However, the details of AS that occurs in ISGs, especially during the late response period, are not well studied. Furthermore, these analyses often focus only on AS at the level of exon splice junctions, not at the entire isoform level due to the limitations of RNA sequencing (RNA-seq) data based on short-read sequencing. To identify the functional and dynamic consequences of IFN responses brought by AS, comprehensive characterization of full-length sequences of isoforms in ISGs is required.

In this study, we applied the PacBio isoform sequencing (Iso-Seq) platform to generate accurate annotations using IFN-I-stimulated B cell lines and examined the detailed time course profiles of isoform switching to understand the impact of AS on ISG function in IFN-I responses. To address this, we predicted protein domains and translation efficiency of all isoforms and discovered that the IFN-I response is regulated not only at the expression level of transcripts but also at the translational level (including changes in protein structures) of transcript isoforms. Furthermore, AS can terminate IFN-I responses by switching intact isoforms to those lacking functional domains. Our data provide deep insight into the regulation of innate immune responses and an understanding of disease mechanisms.

## Results

### Full-length transcriptome profiles in IFN-I-stimulated B cell lines

**Figure 1:**
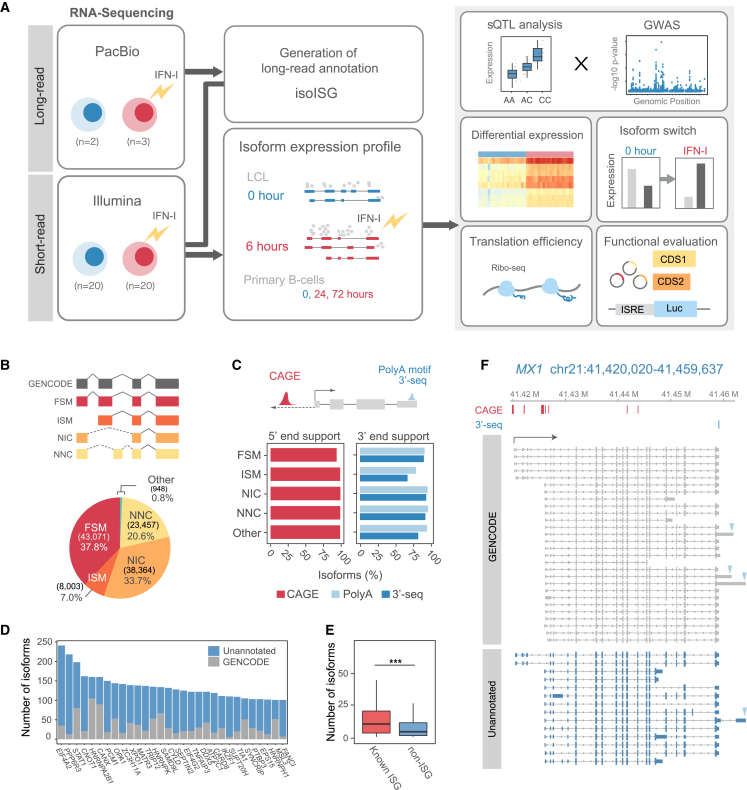
Transcriptome profiling of IFN-I-stimulated LCLs by long-read sequencing (A) Overall schematic workflow of the study design. This figure illustrates the main steps: generating the isoISG annotation from long-read data, quantifying short-read RNA-seq data using isoISG, and performing downstream analyses on the expression data. For the DEG analysis, 20 paired-end RNA-seq samples were used for both unstimulated and stimulated conditions. The sQTL analysis involves a total of 94 samples for both unstimulated and stimulated conditions; refer to Figure S6 for a detailed sQTL analysis workflow. (B) Top: the SQANTI structural categories of newly determined isoforms from stimulated and unstimulated samples. Splice junctions that are not annotated by GENCODE are depicted as dashed lines. Bottom: pie chart showing the number and proportion of structural categories. (C) Percentage of isoforms with CAGE-supported 5′ end and 3′-Seq-supported 3′ end, across each structural category. (D) The top 30 genes with the highest number of detected isoforms. (E) The numbers of isoforms per gene. The list of known ISGs was defined by GSEA. Non-ISGs are genes that were not known as ISGs. ∗∗∗*p* < 0.001 (Wilcoxon rank-sum test, two-sided). (F) MX1 isoforms identified in isoISG. Dark blue and gray represent unannotated and annotated isoforms, respectively. Triangles indicate the positions of poly(A) motifs detected by SQANTI3.

To accurately reconstruct full-length transcriptome profiles of ISGs, we performed RNA-seq on human B lymphocyte cell lines (LCLs) using the PacBio Iso-Seq platform. This included both unstimulated (*n* = 2) and IFN-α2-stimulated samples for 6 h (*n* = 3) (Figure 1A; Table S1). Over 31.7 million circular consensus sequencing (CCS) reads were produced using the single-molecule real-time PacBio Sequel II/IIe system (Figure S1A). After extracting full-length reads, complete with the 5′ and 3′ primers and poly(A) sequence, and subsequent refinement, we obtained HiFi reads with a Q30 accuracy of ≥99.9%. We then collapsed the data and combined the results for further quality control using SQANTI3,^16^ resulting in an Iso-Seq dataset with 251,134 unique reads (Figure S1A). To enhance the accuracy of our transcriptome annotations, we integrated short-read RNA-seq data within the SQANTI3 quality control process to confirm the presence of supporting evidence for the novel splice junctions identified in the Iso-Seq dataset. This validation process led to the compilation of a comprehensive annotation, called isoforms of ISGs (isoISG), comprising 113,843 transcripts. To assess the comprehensiveness of our isoISG annotations, we compared the splice junctions identified in our long-read Iso-Seq dataset with those found in the LCL short-read sequencing data. At the isoform level, 97% of the isoforms in isoISG contained splice junctions that are supported by three or more uniquely mapped short-reads. At the junction level, coverage for each splice junction within isoISG was assessed across short-read samples, revealing that on average, 95.8% of the junctions were covered by each short-read dataset (Figure S1B). Conversely, across these short-read datasets, an average of 73.5% of all identified splice junctions in each sample were found to be covered by our isoISG annotations (Figure S1C).

Each isoform was then classified by finding the best-matching transcripts in the reference annotation (GENCODE version 44) using SQANTI3 (Figure 1B). SQANTI3 classifies isoforms by comparing splice junctions with those of the reference transcript isoforms. When all splice junctions of the query isoform are perfectly matched to the reference transcript, it is categorized as known (full splice match [FSM]). All query isoforms that are not categorized to FSM are novel. Internal splice junctions that match known splice junctions (incomplete splice match [ISM]), the query isoform contains a new combination of known splice sites novel in catalog (NIC), the query isoform contains at least one new donor or acceptor site novel not in catalog (NNC). Overall, 37.8% of our isoforms aligned with known GENCODE annotations. The remainder, making up 62.2%, were categorized as unknown: NIC at 33.7%, NNC at 20.6%, ISM at 7.0%, and Other at 0.8% (Figure 1C). Most isoforms in FSM and ISM were found to match with transcripts annotated as protein coding in Ensembl/GENCODE biotypes (96.6% of FSM and 98.5% of ISM; Figure S1D). A significant proportion of isoforms across various structural categories in the isoISG annotation are supported by FANTOM cap analysis of gene expression (CAGE) for 5′ ends and 3′-Seq for 3′ ends, underscoring the reliability of the annotation. When comparing LCLs, with and without IFN-I stimulation, the distribution of certain isoform categories like NNC and FSM remained consistent (Figure S1E). Notably, while 89% of genes were shared between the stimulated and unstimulated LCL groups, only 51% of isoforms were present in both conditions. For isoforms in structural categories other than FSM (e.g., NIC, NNC, ISM), the percentage shared between the two conditions was even lower, reflecting changes in the transcript landscape under different IFN-I stimulation conditions (Figure S1F).

To understand isoform diversity, we first investigated the number of isoforms per gene and found that some genes had over 100 isoforms (Figure 1D). Following this, we examined the isoform profile of ISGs by comparing the number of isoforms between known ISGs (*n* = 156) and non-ISGs (*n* = 11,197) (known ISGs were defined by the gene set enrichment analysis [GSEA] hallmark ISG list^17^) (Figure 1E). The number of isoforms per gene was high in the ISGs compared to the non-ISGs (median number: 11 vs. 5.0, *p* = 1.8 × 10^−9^; the median percentage of novel isoforms was similar between known ISGs and non-ISGs: 53.0% vs. 52.5%). For example, more than 42 isoforms (14 novel) were detected in the *MX1* gene (Figure 1F). This set of long-read isoform annotations, isoISG, is the core dataset of the present study, which enabled us to accurately investigate changes in isoform expression and the associated AS events upon IFN-I stimulation.

### Changes in transcriptome expression and function during the initial IFN-I response

To examine the variation in IFN responses among individuals at an isoform level, we obtained additional short-read RNA-seq data of LCLs. We compared short-read RNA-seq with long-read sequencing techniques (Oxford Nanopore Technologies [ONT] and PacBio) by analyzing isoform expression levels in LCLs. Short-read sequencing showed a high correlation in isoform expression, suggesting its effectiveness in capturing isoform expression patterns (Figures S1G–S1I).

**Figure 2:**
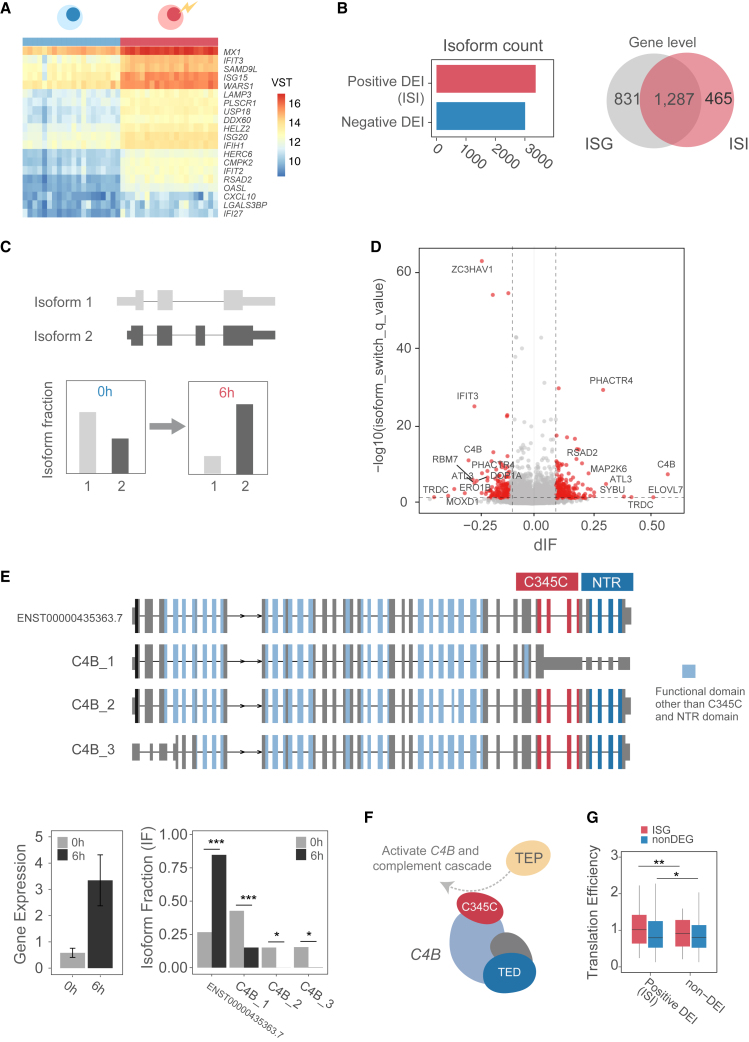
Isoform switch analysis of LCLs in the initial response to IFN-I (A) Heatmap of the top 20 ISGs. Fill color represents normalized count data (variance stabilizing transformation [VST]). (B) Bar graph showing DEI between unstimulated and stimulated samples (left). Positive DEI is defined as ISI. (Right) A Venn diagram representing the overlap of ISGs and DIU isoforms at the gene level. (C) Schematic of isoform switching under two conditions. Unstimulated (IFN-I 0 h) and IFN-I-stimulated (6 h). (D) Volcano plot of dIF and isoform switch q value. Red dots indicate significant isoform switching. q values were calculated using isoformSwitchTestDEXSeq with Benjamini-Hochberg correction. (E) Example of isoform switching in the *C4B* locus. Top: structure of *C4B* isoforms with differing protein domains highlighted. Other domains are in light blue. Bottom: average gene-level expression in unstimulated and stimulated samples (left) and respective average IF (right). ∗FDR < 0.05; ∗∗∗FDR < 0.001 (EdgeR was used for expression analysis and the Mann-Whitney two-sided test was used for IF analysis). Error bars, 95% confidence intervals. (F) Schematic representation of *C4B* interactions with TEPs. The C345C domain interacts with TEPs to activate *C4B* itself and the subsequent complement cascade. (G) Translation efficiency of DEIs calculated by ORQAS. Isoforms are categorized into those with increased expression (positive DEI) and those without expression change (non-DEI). ∗∗FDR < 0.001 (two-way ANOVA, *n* = 676). See also Figure S5.

Following this analysis using data from unstimulated LCLs, we further analyzed LCL-derived RNA-seq data from 20 individuals with/without 6 h of IFN-α2 stimulation, using the isoISG (Figure 1A). Differentially expressed gene (DEG) analysis, which examined the expression level of the entire gene, identified 4,816 DEGs (false discovery rate [FDR] < 0.05), and confirmed that 87.5% of the known ISGs (77 out of 88 genes) were indeed upregulated after 6 h of IFN-α2 stimulation (Figure 2A); we thus defined the positively regulated DEGs (*n* = 2,114) as the ISGs in the present study. We next investigated expression changes at the isoform level in response to IFN-α2 and identified 6,370 differentially expressed isoforms (DEIs) (FDR < 0.05), 3,364 isoforms of which were upregulated (defined as IFN-stimulated isoforms [ISIs]) (Figure 2B, left). These ISIs belonged to 1,748 genes; notably, 465 of the ISI genes did not overlap with the ISG dataset (Figure 2B, right). This suggested that isoform switching, rather than upregulation of gene expression, instigated by AS may be prominent in some genes and that previous studies focusing on ISGs might have underestimated isoform switching during the IFN-I response. Therefore, to further examine isoform usage in detail, we performed a differential isoform usage (DIU) analysis using IsoformSwitchAnalyzeR^18^ (Figure 2C). DIU analysis can determine isoform switches that occur in response to IFN-I, regardless of the changes in overall gene expression. We identified 1,323 DIU isoforms (belonging to 990 genes) with a significant difference in isoform fraction (dIF) in a gene (absolute dIF > 0.05, FDR < 0.05; Figure 2D), and only 33.0% of the DIU isoforms overlapped with ISGs (Figure S2A). This implied that altering specific isoform usage, independent of overall gene expression, also had an impact during the initial 6 h of IFN response.

Among the ISGs exhibiting a DIU effect, complement factor 4 (*C4B*) is known as an essential component in the complement system and destroys foreign pathogens,^19^ and its copy-number variation has been reported to be associated with the risk of developing SLE, Sjogren’s syndrome, and schizophrenia.^20^ While the unannotated isoform in GENCODE, C4B_1, was the major transcript isoform in unstimulated cells, an isoform switch occurred after IFN-I stimulation, with the GENCODE isoform ENST00000435363.7 becoming the dominant isoform (Figure 2E). As the C4B_1 lacks the C345C domain, which is important for *C4B* activation due to binding to thioester-containing proteins (TEPs; Figure 2F), this isoform switch after IFN-I stimulation also altered the function of *C4B* by producing a functional full-length isoform (ENST00000435363.7), and in turn, its translated protein. Therefore, *C4B* may gain function after IFN-I stimulation at both the transcript and protein levels.

The case of *C4B* suggests that IFN-I stimulation may regulate the function of ISIs at the translation or protein level by upregulating specific isoforms or/and switching isoforms (DIU). To understand isoform functional changes, we first focused on translation efficiency. We categorized isoforms into two groups: those with increased expression (positive DEI, or ISI) and those with unchanged expression (non-DEI). Using ORQAS^21^ on the LCL RiboSeq dataset,^22^ we found that isoforms with increased expression had a higher translation efficiency than those without expression changes (*p* value: 1.3 × 10^−2^) (Figure S2B). This increase was even more pronounced within the context of DEGs, particularly ISGs (adjusted *p* value: 1.3 × 10^−3^) (Figure 2G). These findings suggest that upregulation at both the isoform and gene levels plays a crucial role in modulating the translational landscape in response to IFN-I stimulation.

Next, we examined the types of AS events underlying isoform switching upon IFN-I stimulation and found that gain and loss of three AS events were significantly different: alternative transcription terminal site (aTTS), alternative transcription start site, and alternative 3′ end donor site (A3) (FDR < 0.05) (Figures S2C and S2D). Among these, the most significantly increased event was aTTS (FDR = 6.5 × 10^−54^). In addition, we predicted functional consequences of isoform switching among DIUs. This analysis involved comparing the most increased and decreased isoforms within each gene, thereby identifying the consequences of isoform switching. We then evaluated the types of functional changes enriched across the entire DIU gene set (710 genes) after 6 h of IFN-I stimulation. The results indicated a notable increase in isoforms with shorter 3′ UTR sequences in the DIU genes (Figure S2E). This shows that in the initial phase of IFN-I stimulation (6 h), AS events mainly affect gene function through changes in 3′ UTR sequences via aTTS. A previous study using transcript isoforms in polysomes sequencing (TrIP-seq) showed a negative correlation between the length of 3′ UTRs and the number of polysomes associated with isoforms,^23^ indicating that isoforms with shorter 3′ UTRs have higher translational activity. However, when directly comparing translational efficiency among isoforms with varying 3′ UTR lengths within the DIU gene set, no significant difference was observed. In contrast, a significant difference in translation efficiency was noted for DIU genes classified as ISGs (*p* value = 2.7 × 10^−2^).

### Dynamic profiling of isoform switching induced by IFN-I in primary B cells

**Figure 3:**
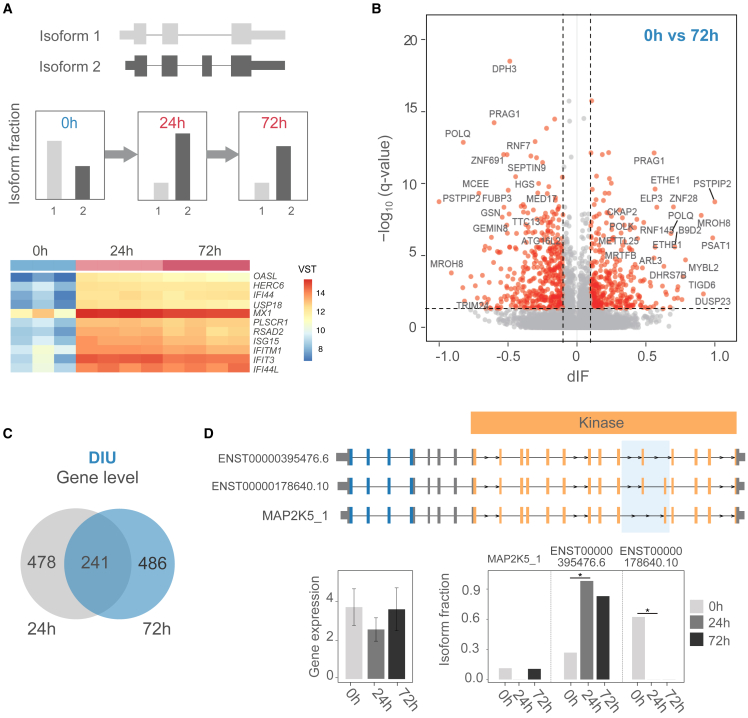
Dynamic changes in isoform usage alter gene function in primary B cells (A) Schematic of isoform switching over two time points after IFN stimulation. Unstimulated (0 h), IFN-stimulated (24 and 72 h). The top 20 ISGs are shown in the heatmap. Fill color represents normalized count data (VST). (B) Volcano plot of dIF and isoform switch q value between unstimulated samples and sample stimulated for 72 h. Red dots indicate dIFs of genes with significant isoform switching. q values were calculated using isoformSwitchTestDEXSeq with Benjamini-Hochberg correction. (C) Venn diagram of gene-level overlap of DIU isoforms detected in two comparisons between unstimulated (0 h) and stimulated samples (24 and 72 h). (D) Example of isoform switching in the *MAP2K5* locus. Top: *MAP2K5* isoform structures with the proline-rich region in light blue. Bottom: average gene-level expressions in unstimulated and stimulated (24 and 72 h) samples (left) and respective average IFs (right). ∗FDR < 0.05 (Mann-Whitney two-sided test). Error bars, 95% confidence intervals.

To further examine dynamic changes in isoform switching until the late phase of IFN responses using isoISG, we analyzed short-read RNA-seq data of primary B cells^24^ (11 samples in total, 3 unstimulated [0 h], 4 samples each at 24 and 72 h post-stimulation) (Figure 3A). To assess the applicability of the isoISG annotation derived from LCLs for quantifying isoform expression in primary B cells, we evaluated the concordance of splice junctions between these datasets. Our analysis revealed that approximately 77%–80% of splice junctions in primary B cell RNA-seq data were covered by our isoISG annotations (Figure S3A), a coverage rate comparable to that observed in LCLs (average 73%–74%) (Figures S1A and S1B). Furthermore, 85%–87% of splice junctions in isoISG were covered by short-read RNA-seq data from primary B cells (Figure S3B). This significant overlap demonstrates the robustness of our isoISG annotation for analyzing primary B cell data, thereby supporting its use in our subsequent analyses.

We then utilized isoISG to identify two DIUs by comparing unstimulated and stimulated samples and examined the isoform switches (Figure 3B) in primary B cells. A total of 719 and 727 DIUs belonging to 623 and 637 genes, respectively, were detected in the unstimulated-stimulated comparisons (0 h vs. 24 and 72 h). More than half of the DIU genes in the two comparison groups did not overlap, indicating that time course alterations in isoform usage occurred (Figure 3C). Among the isoform switches we identified, one interesting example of dynamic time course change was observed in mitogen-activated protein kinase kinase 5 (*MAP2K5*) when comparing 0 h vs. 24 h (Figure 3D). The *MAPKK* gene family, to which *MAP2K5* belongs, is known to play a pivotal role in immune cell functions, including proliferation, differentiation, and the regulation of cytokine production.^25^ We identified isoforms that lack segments of the kinase domain essential for these activities (*MAP2K5_1* and ENST00000395476.6, respectively).^26^ These missing segments reside within the proline-rich region, a domain known to facilitate protein-protein interactions and signaling. The isoform containing the intact kinase domain ENST00000178640.10 showed a significant increase in its expression ratio at 24 h post-stimulation, suggesting a time-dependent regulatory mechanism in response to immune activation (Figure 3D).

**Figure 4:**
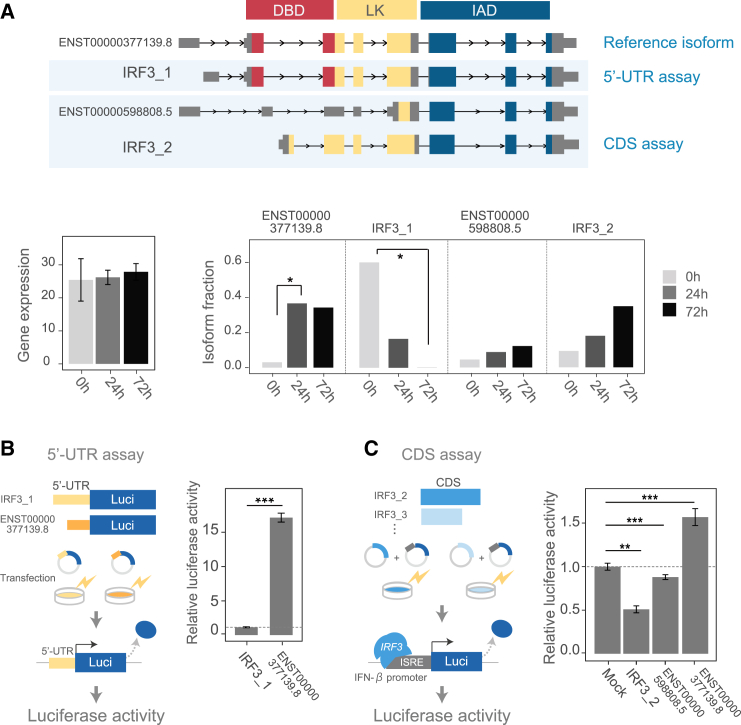
Experimental validation of functional differences among *IRF3* gene isoforms using UTR and CDS assay (A) Example of *IRF3* isoform switching. Top: *IRF3* isoform structures, with those used in the functional assay in light blue boxes. Protein domain: DBD, linker region (LK), and IAD. Bottom: average gene-level expression in unstimulated (0 h) and stimulated (24 and 72 h) samples (left) and respective average IFs (right). ∗FDR < 0.05 (Mann-Whitney two-sided test). Error bars, 95% confidence intervals. (B) Comparison of isoform expression (relative luciferase activity) using the 5′ UTR assay. ∗*p* < 0.05 (t test, two sided). Means and SDs are shown for four technical replicates. Data represent a representative experiment from three independent experiments. Luci, luciferase reporter gene. (C) Comparison of isoform promoter activity (relative luciferase activity) using the CDS promoter assay. Means and SDs are shown for four technical replicates. Data represent a representative experiment from three independent experiments. ∗∗FDR < 0.01; ∗∗∗FDR < 0.001 (t test, two sided).

Another example of time course isoform switching was *IRF3*, which is a central transcription factor involved in regulating IFN-I signaling. After IFN-I stimulation, isoforms with different 5′ UTR sequences (IRF3_1 and ENST00000377139.8) were switched (Figure 4A). The isoform changes observed in *IRF3* suggested that the functional consequences brought by the isoform switch would play a substantial role in triggering and terminating the IFN response. To substantiate the expression and functional significance of the novel IRF3 isoforms, we first confirmed the presence of novel *IRF3* isoforms (*IRF3_1* and *IRF3_2)* through ONT RNA-seq in LCL (Figure S3C). Next, to assess whether individual isoforms have different functions, we conducted experimental validation using *IRF3*. We compared two isoforms with different 5′ UTR but the same full-length open reading frames (ORFs) using the 5′ UTR assay (Figure 4B, left). Compared to the 5′ UTR of IRF3_1, the 5′ UTR of ENST00000377139.8 displayed significantly higher protein levels as evaluated by luciferase activity (Figure 4B, right). Together with the dynamic changes in expression levels of these isoforms (decrease of IRF3_1 and increase of ENST00000377139.8), the proportion of isoforms with higher protein translation would increase after IFN-I stimulation. Next, the function of isoforms with different ORFs was examined. Because the DNA binding domain (DBD) of *IRF3* binds to ISREs of ISGs, including IFN-β, we compared the functions of protein isoforms using their ability to activate IFN-β using coding sequence (CDS) assay^27^ (Figure 4C, left). We compared two isoforms lacking functional domains with the full-length ORF isoform (ENST00000377139.8). The results show that the isoform lacking part of the DBD and the IRF association domain (IAD) that mediates IRF dimerization (IRF3_3) lacked the ability to activate the IFN-β promoter. Notably, isoforms that lack most of the DBD (IRF3_2 and ENST00000377139.8) suppressed the IFN-β promoter, indicating a dominant-negative effect of the domain-deficient isoform on endogenous *IRF3* proteins expressed in HEK293 cells (Figure 4C, right). These results suggest that the isoforms have distinct functions, their isoform ratio changes upon IFN-I stimulation, and, as a result, the collective function of the gene may change over time.

### Intron retention with domain loss dynamically changes gene function in the late phase of IFN-I stimulation

To further characterize the functional consequence of isoform changes during late-phase IFN-I responses, we examined the types of isoform switches among DIUs in primary B cells. Intron retention (IR) was significantly increased after 24 and 72 h of stimulation (FDR < 0.05; Figure S4A). Analysis of the consequences of these isoform switches showed an increase in 3′ UTR length and nonsense-mediated mRNA decay (NMD)-sensitivity status (Figure S4B). The increase in NMD-sensitivity status might be mediated by IR due to the frequent introduction of premature termination codons (PTCs) that may promote NMD of mRNA.^28^^,^^29^^,^^30^ Even if these isoforms with PTCs escape NMD, the translated protein isoforms may lose functional domains; therefore, IR may have negative effects on gene function at both the transcript and protein levels.

**Figure 5:**
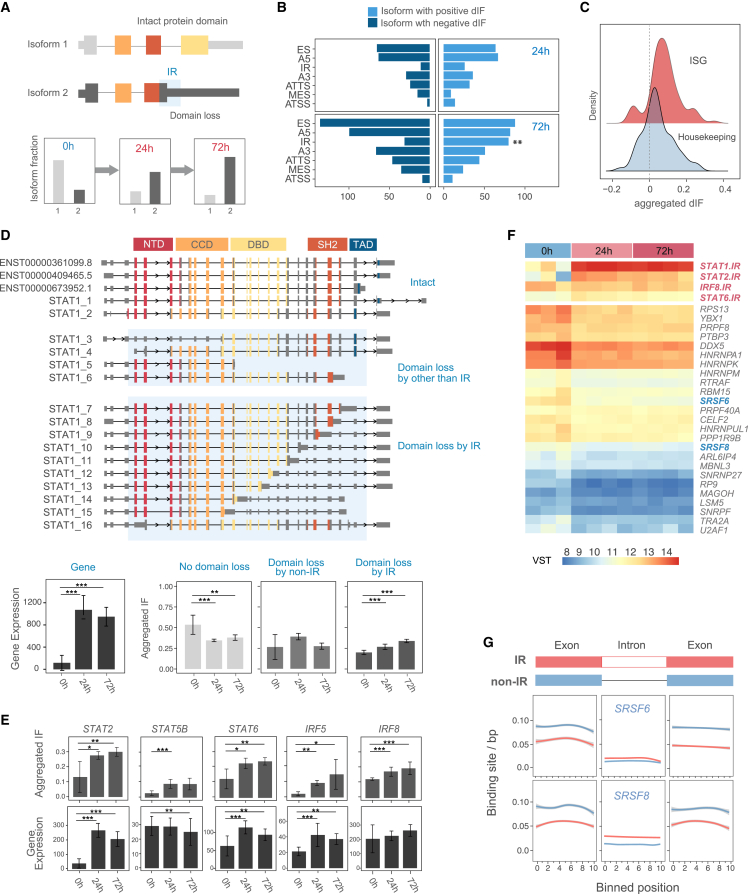
Impact of IR isoforms during the late phase of the IFN-I response (A) Schematic of isoform switching with IR events over two time points after IFN-I stimulation. Unstimulated (0 h), IFN-I-stimulated (24 and 72 h). Isoform 2, due to an IR event, acquires an early stop codon that truncates the protein, leading to the loss of the third protein domain, represented in yellow. (B) The number of domain loss events by AS types. ∗∗FDR < 0.01 (chi-square test, *n* = 1,259). ES, exon skipping; A5, alternative 5′ end donor site; A3, alternative 3′ end acceptor site. (C) Comparison of aggregated dIFs between ISGs and housekeeping genes. (D) Example of *STAT1* isoforms with IR events. Top: two reference *STAT1* isoform structures (top); novel isoforms with IR events boxed in light blue. Bottom: average aggregated expression in unstimulated and stimulated (24 and 72 h) samples at the gene level (left) and isoform level (right; average aggregated IF). ∗∗∗FDR < 0.001 (Mann-Whitney two-sided test). Error bars, 95% confidence intervals. (E) Aggregated IF of additional ISGs. ∗FDR < 0.05; ∗∗FDR < 0.01; ∗∗∗FDR < 0.001. (F) Heatmap showing splicing factor genes with strong negative correlations (|r^2^| < 0.9), with expression of *STAT1* (*STAT2.IR*), *STAT2* (*STAT2.IR*), *STAT6* (*STAT6.IR*), and *IRF8* (*IRF8.IR*) isoforms with IR events. Genes with IR and SRSF genes are shown in red and blue, respectively. (G) Examples of number of binding sites per base pair of SRSF genes for exons and introns with/without IR events. The x axis represents relative binned position along each exon/intron. All differences in binding sites between exons and introns with/without IR events are significant difference (FDR < 0.001).

We thus hypothesized that IR has a significant impact on gene function (Figure 5A). To address this, we focused our investigation on isoforms with domain loss by IR, since it was clear that domain loss was an indication of altered isoform function. We first identified isoforms in isoISG that lost domains by various types of AS events. Then, we separately counted the number of domain loss events in isoforms whose fraction increased/decreased after IFN-I stimulation (Figure 5B). In the isoforms with increased fraction (dIF > 0.05) upon IFN-I stimulation (72 h), more domain loss was detected by IR compared to non-IR splicing events (FDR = 9.3 × 10^−4^, chi-square test). This trend was similar when isoforms with smaller dIF (>0.01) were evaluated (2.2 × 10^−4^, chi-square test; Figure 5B). These results suggested that the IR events had a significant impact on the function of ISGs. In fact, increased fractions of IR isoforms upon IFN-I stimulation (0 h vs. 72 h) in ISGs was significantly higher than that of housekeeping genes (*p* < 10^−5^ Wilcoxon rank-sum test; Figure 5C), suggesting that IR events in ISG play an important role in diminishing the function of ISGs.

Notably, *STAT1* is the key mediator of type I IFN-I signaling and has many IR isoforms (Figure 5D), most of which are unannotated in GENCODE. The aggregated fraction of IR isoforms in *STAT1* increased from 0.19 (0 h) to 0.27 (24 h) and 0.34 (72 h) after IFN-I stimulation (Figure 5D). Meanwhile, the aggregated fraction of isoforms without domain loss decreased with time after IFN-I stimulation. Collectively, the function of *STAT1* would be suppressed until the late phase of the IFN-I response, which cannot be evaluated by simply looking at *STAT1* gene-level expression. Interestingly, other members of the STAT gene family, in particular *STAT2*, *STAT5B*, and *STAT6*, showed significant increases in IR isoforms over time, as observed in *STAT1* (Figures 5E and S4C).

To investigate the mechanism by which such IR events are induced, we examined the splicing factors that correlate strongly with the time course expression of IR isoforms of ISGs (Figure 5F). Because a previous study showed that knockdown of serine/arginine (SR)-rich splicing factors (SRSF) increased IR events,^31^ we focused on the splicing factors that showed negative correlation with the expression of IR isoforms. Among them, *SRSF6* and *SRSF8* negatively correlated with the IR isoform expression (|r^2^| > 0.9; Figure 5F). Comparison of the number of SRSF family binding sites in exons and introns with and without IR events revealed a significant difference (FDR < 0.001). In particular, the number of SRSF binding sites was lower in exons and higher in introns with IR events (Figure 5G), suggesting that IR events are not random but more likely to occur in specific exon-intron regions under the regulation of splicing factors.

### Isoform switching across various cell types and IFN-I stimulations

Based on our observations in B cells, we aimed to examine the impact of IFN-I on isoform switching in various immune cell types and under different IFN-I stimulations. To this end, we employed short-read RNA-seq datasets from dendritic cells (DCs) stimulated with IFN-β.^32^^,^^33^ Specifically, in DCs stimulated for 4 h (*n* = 20 each), we observed a similar change (aTTS gain) in line with the initial response of B cells to IFN-I (Figure S5A). Notably, *C4B* exhibited an isoform switch identical to the early response to IFN-I observed in LCLs (Figure S5B, top). Conversely, *IRF3* presented a distinct isoform switching from that observed in the early IFN-I response of LCL (Figure S5B, bottom). At the 12-h time points in DCs (*n* = 3 each), there was a significant increase in IR and a decrease in A5 splice site usage, consistent with the patterns of late-phase IFN-I response in B cells (Figure S5C). We also acquired additional data using a monocytoid cell line (THP-1 cells) treated with IFN-α2 and IFN-β for 6 h (*n* = 3 each) using ONT RNA-seq. We observed statistically significant AS changes characterized by 3′ UTR shortening in response to IFN-α2 (*p* = 0.033; Figure S5D). Although 3′ UTR shortening in response to IFN-β lacked statistical significance, the overall trends for both IFN-α2 and IFN-β were similar, aligning with the initial IFN-I response observed in short-read data. Overall, the temporal dynamics of IFN-I responses might represent a universal characteristic across different immune cell types, with some variations among the cell types. This indicates a fundamental aspect of the immune response to IFN-I stimulation.

### Mining disease-associated isoforms via quantitative trait loci analysis

**Figure 6:**
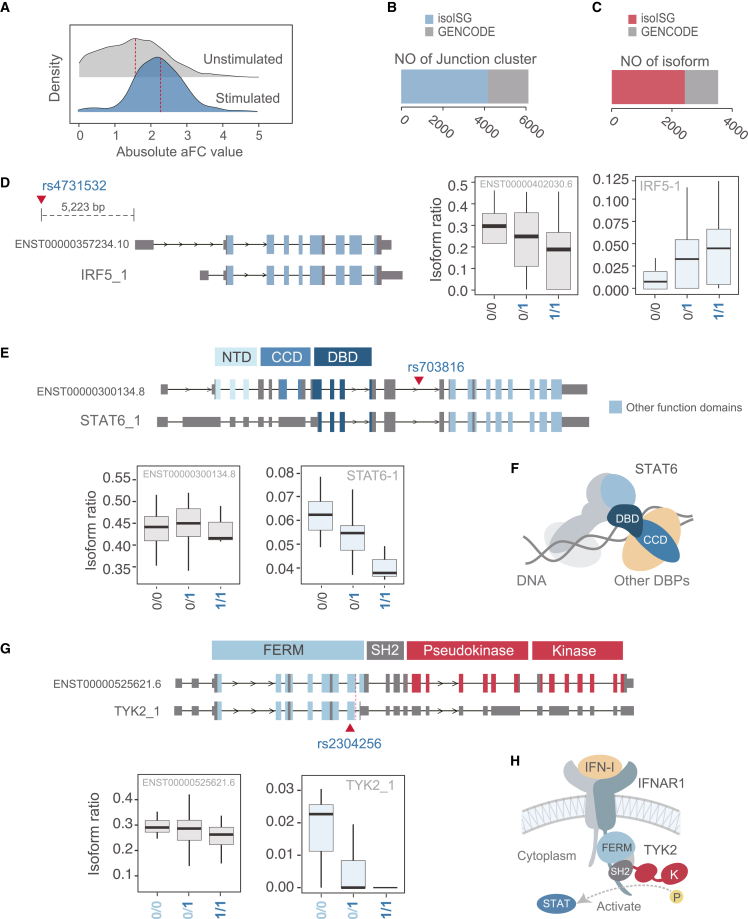
Mining disease-related isoforms in isoISG via QTL analysis (A) Effect size comparison between sQTLs of stimulated and unstimulated LCLs. The effect size was estimated by calculating log allelic fold change (aFC). *p* < 0.001 (Wilcoxon rank-sum test, one sided). The aFC was transformed to absolute values, with the median (red dotted line). (B) The number of junction clusters with significant sQTLs identified using the LeafCutter pipeline. The number of junctions included only in isoISG (light blue) and GENCODE (gray). (C) The number of isoforms with significant sQTLs identified by ir-QTL. Isoforms included only in isoISG and GENCODE are shown in red and gray, respectively. (D) An example of novel splice junctions with a significant sQTL in the *IRF5* locus. The IFs by corresponding genotype are shown at right. The risk allele (1) is highlighted in bold blue. The IF of a reference (ENST00000402030.6) that lacks a significant ir-QTL is shown for comparison. Only the top isoform with the highest RTC score is shown for the significant sQTL. A red triangle indicates the location of the sQTL. (E) An example of a novel isoform with a significant sQTL in the *STAT6* locus. The IFs by corresponding genotype are shown at lower right. The risk allele for asthma (1) is highlighted in bold blue. The IF of a reference (ENST00000300134.8) that lacks a significant ir-QTL is shown for comparison. A red triangle indicates the location of the sQTL. (F) Schematic representation of interactions between the CCD domain of *STAT6* and other DBPs. (G) An example of isoforms with a significant sQTL in the *TYK2* locus. The IFs by corresponding genotype are shown at bottom. The IF of a reference (ENST00000525621.6) that lacks a significant ir-QTL is shown for comparison. A red triangle indicates the location of the sQTL. Risk alleles for immunological disease (1) and severe COVID-19 (0) are blue and black, respectively. (H) Schematic representation of interactions between the FERM, SH2, and kinase domains of *TYK2* with *IFNAR1* and STAT. P, phosphate; K, kinase domain.

AS events can be influenced by the presence of genetic variants, known as splicing quantitative trait loci (sQTL). Certain sQTL cause complex diseases by altering gene function.^34^ Therefore, we conducted sQTL analyses to identify disease-associated isoforms and assessed the co-localization of sQTLs and genome-wide association study (GWAS) variants^35^ (see detailed workflow in Figure S6). Initially, we performed junction-based sQTL analysis using LeafCutter^36^ on IFN-I-stimulated and unstimulated LCLs from the Japanese population (*n* = 94 each) (Figures 1A and S6). Using whole-genome sequence data of samples from the 1000 Genomes Project, we identified 6,010 and 2,438 sQTLs in the IFN-I-stimulated and unstimulated samples, respectively (FDR < 0.05). The effect size of the sQTLs on the ISG genes, which was significant in both stimulated and unstimulated samples, was significantly larger in the stimulated sample than in the unstimulated sample (*p* < 0.001, Wilcoxon rank-sum test; Figure 6A), indicating that IFN-I stimulation enhances sQTL effects.

As we confirmed that 47.4% of the junctions (3,293/6,943) with significant sQTLs were present in the isoISG isoforms (Figure 6B), we performed isoform-ratio QTL (ir-QTL) analysis using the isoISG annotations. ir-QTL can distinguish the sQTL effects of isoforms sharing the same junctions identified in the junction-based sQTL. The ir-QTL analyses identified 2,570 ir-QTL isoforms, 55.1% of which were novel isoforms (Figure 6C). To assess the involvement of isoISG isoforms with significant sQTL effects in complex traits, we evaluated the co-localization of sQTL and GWAS loci in the GWAS Catalog using regulatory trait concordance (RTC).^37^ A total of 1,023 sQTLs were co-localized with GWAS loci (RTC ≥ 0.9) for at least one trait. Among the IFN-I signaling pathway genes, a SNP (rs4731532) had an ir-QTL effect for an *IRF5* isoform (*IRF5_1*, unannotated in GENCODE) and was co-localized with GWAS signals linked to a range of autoimmune diseases, including systemic sclerosis, Sjögren’s syndrome, and RA (Figure 6D). We also found an ir-QTL (rs703816) for an isoform of *STAT6* (STAT6_1) that co-localized with the asthma signal (Figure 6E). This isoform lacks the coiled-coil domain (CCD), which is important for interactions with several DNA binding proteins (DBPs) such as IRF genes (Figure 6F), and the expression level of this isoform was decreased with the number of disease risk alleles. Furthermore, we identified a significant co-localization with GWAS signals in a SNP (rs2304256) for the *TYK2* gene (Figure 6G), which was associated with multiple traits, including type 1 diabetes mellitus, SLE, primary biliary cirrhosis, and psoriatic arthritis. Significant ir-QTL effects were observed for *TYK2_1*, whose CDSs differed from the GENCODE reference isoform (ENST00000525621.6). The isoform *TYK2_1* was upregulated at the risk allele (Figure 6G), which lacked the SH2 domain that mediates the interaction with the cytoplasmic tail of the IFN receptor type I gene (*IFNAR1*) (Figure 6H). Intriguingly, the co-localization of this ir-QTL was also observed for the GWAS signal of severe COVID-19 (Host Genetic Initiative release 7)^38^ (Figure 6G), although the risk allele was inverse compared to that for autoimmune diseases.

## Discussion

We developed an isoform catalog of B cell lines, isoISG, to define isoforms upon IFN-I stimulation by using long-read sequencing. Our pipeline has identified a total of 163,747 isoforms, over 80% of which were unannotated in GENCODE. Of note, the majority of the novel isoforms were enriched in ISGs as compared to non-ISGs, suggesting that the immune system has acquired its function to attack various foreign pathogens by increasing the function of existing genes upon stimulation through AS. Indeed, a recent study demonstrated that splicing isoforms of the *OAS1* gene, an ISG, determined the outcome of COVID-19.^39^^,^^40^

We used our isoISG to show how gene function is affected by time course changes in isoform expression after IFN-I stimulation. In the initial response to IFN-I stimulation, isoform switching occurred to upregulate isoforms with high translational efficiency, and many of these were accompanied by changes in the UTR. We experimentally validated that the 5′ UTR switch of *IRF3* would increase the fraction of isoforms with greater protein production. Our RiboSeq and RNA-seq analyses indicated no significant translational efficiency difference between isoforms with varying 3′ UTR lengths. This observation contrasts with TrIP-seq studies suggesting that shorter 3′ UTRs are typically associated with higher translational activity.^23^ However, we found that ISI and ISG statuses are more potent determinants of translational efficiency after IFN-I stimulation. These findings highlight a complex regulatory landscape of translation, in which multiple factors, including isoform context and cellular environment, play crucial roles under IFN-I stimulation. Moreover, as seen in the case of *C4B*, some of the isoform switches in ISGs would trigger the function of genes by transforming the structure of proteins from an inactive (a protein isoform of *C4B* lacking the 3′ domain) to an active form (a full-length *C4B*). Although most previous studies have focused on the transcript levels of ISG, our finding demonstrated that ISGs gain function upon IFN-I stimulation at the translation and protein levels through AS.

Conversely, in the late phase of IFN-I response (72 h), isoform switching occurred with IR events, which may reduce the function of the ISG genes through domain loss. Notably, IR events were enriched in the STAT genes, which are key players in the IFN-I response, suggesting that IR events may be essential for negative feedback or termination of the IFN-I response. IR events have previously received attention for their role in developmental stages and cell differentiation.^31^^,^^41^ However, the role of IR isoforms, particularly in the immune response, has been underestimated due to insufficient annotation of IR isoforms in the public catalogs, such as the GENCODE annotation, which are widely used in transcriptome analyses of immunology. We found that the expression of SR proteins (*SRSF5*, *SRSF6*, *SRSF8*) showed strong negative correlations with the expression of the IR isoforms. SR proteins were recently shown to be repressed by IFN-I signaling.^42^^,^^43^ Their binding sites were also reported to be enriched in retained introns, and knockdown of *SRSF1* and *SRSF7*, for example, increased IR events.^31^ Therefore, reduced expression of SR proteins following IFN-I stimulation may be responsible for the increased IR events, which counters the positive feedback loop of the IFN-I response.

In addition to demonstrating the importance of dynamic changes in the isoform profile of ISGs, we have shown that genetic variants (sQTLs) exhibit a diversity of isoform profiles in individuals, which may cause various diseases. For example, we found that novel isoforms, which are not registered in GENCODE (*IRF3*, *STAT6*, and *TYK2*), were involved in the pathogenesis of various immunological diseases. An isoform of *TYK2* (TYK2_１) has a FERM domain but lacks a kinase domain. Therefore, this isoform may bind to the cytoplasmic tail of *IFNAR1* but cannot phosphorylate the STAT gene (see Figure 5H). Binding of this isoform to *IFNAR1* may inhibit binding of the full-length *TYK2* isoform, which may result in negative feedback for the IFN-I response. Similarly, a *STAT6* isoform (*STAT6_1*) with an IR event lacked the CCD that interacts with other DBPs (Figure 5F). The lack of binding of this isoform to DBPs, which stabilize binding to DNA, may weaken the transcription factor activity, again, resulting in negative feedback on the IFN-I response. Given that both gene isoforms (*TYK2_１* and *STAT6_1*) are downregulated in the disease risk allele, the failure of the negative feedback mechanism in the IFN-I response may enhance the immune response to foreign pathogens but also increase the risk of disease. Indeed, the converse association observed in the *TYK2* variant with severe COVID-19 may indicate that the reduced immune response due to a higher ratio of *TYK2_１* may result in insufficient responses to the coronavirus. These findings suggest that it is essential to profile dynamic changes in the ratio of splicing isoforms together with gene expression to fully understand the immune response to foreign pathogens, as well as the mechanism of immunological diseases.

### Limitations of the study

The primary limitation is that isoISG is an isoform catalog limited to IFN-α2 stimuli. Since AS events are regulated in a signal-specific manner,^12^^,^^13^ isoISG may not include isoforms induced by stimuli other than IFN-α2. However, since there are many common ISG genes induced by IFN-α and IFN-β, we believe that it is worth using the isoISG for detailed analysis focusing on the ISG genes. This is also the case for the cell specificity of AS. Our isoISG is generated from B cell lines, and it will be necessary to generate annotations for different cell types.

Furthermore, another key limitation identified in our study pertains to the use of RiboSeq as a methodological tool. While RiboSeq has been invaluable for studying translational efficiency, it has demonstrated limited sensitivity in differentiating between isoforms based on UTR lengths. This highlights the necessity for more detailed experimental approaches in future research to fully discern the complexities of isoform-specific translation dynamics. Such enhanced methodologies would allow for a deeper understanding of the nuanced roles of isoforms in regulating gene function under various biological conditions.

## Resource availability

### Lead contact

For further information and requests for resources and reagents, please contact Yuta Kochi (y-kochi.gfd@mri.tmd.ac.jp).

### Materials availability

This study did not generate new unique reagents.

### Data and code availability

All RNA-seq datasets sequenced in this study were deposited in the DDBJ Sequence Read Archive under BioProject accession PRJDB15952 and are publicly available as of the date of publication. All original code and related data have been deposited at github (https://github.com/uedaMT/isoISG) and via Zenodo (https://doi.org/10.5281/zenodo.13282235). Results in this study can be browsed at the University of California, Santa Cruz Genome Browser (https://genome.ucsc.edu/s/UEDA/isoISG). Any additional information or help in reanalyzing the datasets is available from the lead contact upon request.

## Acknowledgments

We thank Kyoko Kobayashi (RIKEN), Takayo Tsuchiura, and Nao Nishida (Tokyo Medical and Dental University) for their technical assistance. This work was supported by the 10.13039/501100001691Japan Society for the Promotion of Science (JSPS) Grant-in-Aids for JSPS Fellows (grant no. 21J00596) (J.I.), Grant-in-Aids for Scientific Research (B) (grant nos. 18H02849 and 22H02597), Grant-in-Aid for Challenging Research (grant no. 21K19501), and Grant-in-Aid for Scientific Research (C) (grant no. JP24K10050) (M.T.U.) from 10.13039/501100001700MEXT, Japan. This work was also supported by grants from Nanken-Kyoten, TMDU (2023-kokusai02), Medical Research Center Initiative for High Depth Omics, TMDU, and the Uehara Memorial Foundation, all awarded to Y.K. Computations were partially performed on the NIG supercomputer at the ROIS National Institute of Genetics.

## Author contributions

M.T.U. conducted the bioinformatics analysis with the help of J.I. and K.Y. Y.K. designed and managed the project. M.S. generated the PacBio Iso-Seq data. F.M. provided part of the short-read RNA-seq datasets. M.T.U. and Y.K. wrote the manuscript. All authors approved the final manuscript.

## Declaration of interests

The authors declare no competing interests.

## STAR★Methods

### Key resources table

**Table:** 

REAGENT or RESOURCE	SOURCE	IDENTIFIER
**Chemicals, peptides, and recombinant proteins**		

RPMI-1640	FUJIFILM Wako Pure Chemical Corporation	Cat# 189-02025
D-MEM (High Glucose) with L-Glutamine and Phenol Red	Wako Pure Chemical Industries	Cat# 044-29765
Fetal bovine serum	Gibco	Cat# 42Q7361K
Penicillin-streptomycin	Wako Pure Chemical Industries	Cat# 168-23191
Recombinant Human IFN-α2 (carrier-free)	BioLegend	Cat# 592702
TRIzol reagent	Thermo Fisher Scientific	Cat#15596018
poly (I:C)	Enzo Life Sciences	Cat# ALX-746-021-M005
Ribo-Zero rRNA Removal Kit	Illumina	Cat# MRZH11124
TruSeq RNA Sample Prep Kits v2	Illumina	Cat# RS122-2001

**Critical commercial assays**		

RNeasy Mini Kit	QIAGEN	Cat#74104
NEBNext Single Cell/Low Input cDNA Synthesis & Amplification Module	New England Biolabs	Cat# E6421L
ProNex Size-Selective Purification System	Promega Corporation	Cat# NG2001
SMRTbell Express Template Prep 2.0	Pacific Biosciences	Cat# 100-938-900
Sequel II Sequencing 2.0 Kit	Pacific Biosciences	Cat# 101-820-200
SMART-seq v4 Ultra Low Input RNA Kit for Sequencing	Takara Bio	Cat# 634891
SMARTScribe Reverse Transcriptase	Takara	Cat# 639538
AMPure XP beads	Beckman Coulter	Cat# A63880
SeqAmp DNA Polymerase	Takara Bio	Cat# 638504
Ligation Sequencing Kit	Oxford Nanopore Technologies	Cat#SQK-LSK110
Ligation Sequencing Kit V14	Oxford Nanopore Technologies	Cat#SQK-LSK114
NEBNext Poly(A) mRNA Magnetic Isolation Module	New England Biolabs	Cat# E7490L
NEBNext UltraDirectional RNA Library Prep Kit for for Illumina	New England Biolabs	Cat# E7420L
pcDNA3.1Directional	Thermo Fisher Scientific	Cat# K490001
pFL-SV40 vector	Addgene	Cat#115352
pEF-BOS-hTLR3-FLAG vector	Dr. Matsumoto, Hokkaido University	N/A
p55C1B-Luc vector	Dr. Fujita, Kyoto University	N/A
pGL4.74 vector	Promega	Cat# E6921
X-tremeGENE HP Transfection Reagent	Roche	Cat# 6366244001
Dual Luciferase Reporter assay system	Promega	Cat#N1630

**Deposited data**		

PacBio Sequel II Iso-Seq data	This paper	DRR477716 (BioProject: PRJDB15952); https://ddbj.nig.ac.jp/search/entry/sra-submission/DRA016394
PacBio Sequel IIe Iso-Seq data	This paper	DRR568661 -DRR568664 (BioProject: PRJDB15952); https://ddbj.nig.ac.jp/search/entry/sra-submission/DRA018714
ONT RNA-Seq	This paper	DRR477708 -DRR477715 (BioProject: PRJDB15952); https://ddbj.nig.ac.jp/search/entry/sra-submission/DRA016393
Short-read RNA-Seq data	This paper	DRR477717 -DRR477830 (BioProject: PRJDB15952); https://ddbj.nig.ac.jp/search/entry/sra-submission/DRA016395
UCSC data browser for isoISG	This paper	https://genome.ucsc.edu/s/UEDA/isoISG
*De novo* isoform annotation (isoISG)	This paper	*De novo* isoform annotation (isoISG)
Human reference genome NCBI build 38, GRCh38	Genome Reference Consortium	https://www.ncbi.nlm.nih.gov/projects/genome/assembly/grc/human/
1000 Genomes Project reference panel (phase3) (http://ftp.1000genomes.ebi.ac.uk/)	(Auton et al., 2015)^44^	https://www.internationalgenome.org/data
GENCODE annotation v39 (GRCh38)	(Frankish et al., 2021)^45^	https://www.gencodegenes.org/
ribo-seq	(Battle et al., 2015)^22^	GEO: GSE61742
Genotype and RNA-Seq dataset of Genetic European Variation in Disease (GEUVADIS)	(Lappalainen et al., 2013)^46^	https://www.internationalgenome.org/data-portal/data-collection EMBL-EBI, E-GEUV-1
NHGRI-EBI GWAS Catalog	(Buniello et al., 2019)^35^	https://www.ebi.ac.uk/gwas/
GWAS summary statistics of COVID-19 release 7 (A2_ALL_leave_23andme, very severe respiratory confirmed COVID vs. population)	(COVID-19 Host Genetics Initiative, 2022)^38^	https://storage.googleapis.com/covid19-hg-public/20201215/results/20210107/COVID19_HGI_A2_ALL_eur_leave_23andme_20210107.b37.txt.gz).
MSigDB hallmark gene set collection (v7.5.1)	(Liberzon et al., 2015)^17^	https://www.gsea-msigdb.org/gsea/msigdb/collections.jsp
refTSS (v4.1)	(Abugessaisa et al., 2019)^47^	https://reftss.riken.jp/datafiles/4.1/human/refTSS_v4.1_human_coordinate.hg38.bed.txt.gz
RNA-Seq (Primary B-cell)	(Wirz et al., 2021)^24^	GEO: GSE118875
TSSclassifier	(Forrest et al., 2014)^48^	https://dbarchive.biosciencedbc.jp/data/fantom5/datafiles/phase1.3/extra/TSS_classifier/TSS_human.bed.gz
FANTOM5 CAGE phase1&2 hg38	(Severin et al., 2014)^49^	https://zenbu-wiki.gsc.riken.jp/zenbu/wiki/index.php/Data_Download

**Experimental models: Cell lines**		

LCL (PacBio Sequel II Iso-Seq, 0h and 6h)	Coriell	GM12878
LCL (PacBio Sequel II Iso-Seq, 0h and 6h)	Coriell	GM19078
LCL (ONT RNA-Seq, 0h and 6h)	Coriell	GM18943
LCL (ONT RNA-Seq, 0h and 6h)	Coriell	GM19066
LCL (ONT RNA-Seq, 0h and 6h)	Coriell	GM19075
LCL (ONT RNA-Seq, 0h and 6h)	Coriell	GM19078
HEK-293T	ATCC	Cat#CRL-1573

**Software and algorithms**		

Guppy (v4.4.1)	https://community.nanoporetech.com/	https://community.nanoporetech.com/
minimap2 (v2.17)	(Li, 2018)^36^	https://github.com/lh3/minimap2
FLAIR (v1.5)	(Tang et al., 2020)^50^	https://github.com/BrooksLabUCSC/flair
SQANTI3 (v5.2)	(Tardaguila et al., 2018)	https://github.com/ConesaLab/SQANTI3
Salmon (v1.10.2)	(Patro et al., 2017)^51^	https://combine-lab.github.io/salmon/getting_started/#obtaining-salmon
RBPmap (v1.2)	(Paz et al., 2014)^52^	http://rbpmap.technion.ac.il/
SignalIP-5.0	(Almagro Armenteros et al. 2019)^53^	https://services.healthtech.dtu.dk/service.php?SignalP-5.0
CPAT (v 3.0.4)	(Wang et al., 2013)^54^	https://sourceforge.net/projects/rna-cpat/files/?source=navbar
IUPred3 (v.3)	(Dosztányi, 2018)^55^	https://iupred3.elte.hu/
Pfam (v35.0)	(Mistry et al., 2021)^56^	http://pfam-legacy.xfam.org/
LeafCutter (v0.2.9)	(Li et al., 2018)^36^	https://github.com/davidaknowles/leafcutter/
STAR (v2.7.3)	(Dobin et al., 2013)^57^	https://github.com/alexdobin/STAR
R (v4.2)	(R Core Team, 2018)	https://www.r-project.org/
peer (v1.0)	(Stegle et al., 2012)^58^	https://github.com/PMBio/peer
polyester (v1.38.0)	(Frazee al, 2015)^59^	https://github.com/alyssafrazee/polyester-release
IsoformSwitchAnalyzeR (v1.8.0)	(Vitting-Seerup et al., 2019)^18^	https://bioconductor.org/packages/release/bioc/html/IsoformSwitchAnalyzeR.html
DRIMSeq (v1.14.0)	(Nowicka and Robinson, 2016)^60^	https://bioconductor.org/packages/release/bioc/html/DRIMSeq.html
edgeR (v3.36.0)	(Robinson et al., 2017)	https://bioconductor.org/packages/release/bioc/html/edgeR.html
QTLtools (v1.3.1)	(Delaneau et al., 2017)^61^	https://qtltools.github.io/qtltools/
BEDtools (v2.29.2)	(Quinlan et al., 2010)^62^	https://github.com/arq5x/bedtools2
cDNA-Cupcake (v27.0.0)	https://github.com/Magdoll/cDNA_Cupcake	https://github.com/Magdoll/cDNA_Cupcake
Fastp (v0.21.0)	(Chen et al., 2018)^63^	https://github.com/OpenGene/fastp
CCS (v5.0.0)	https://github.com/PacificBiosciences/ccs	https://github.com/PacificBiosciences/ccs
Iso-Seq3 (v3.4.0)	https://github.com/PacificBiosciences/Iso-Seq	https://github.com/PacificBiosciences/Iso-Seq
Pychopper (v2.0)	Oxford Nanopore Technologies	https://github.com/epi2me-labs/pychopper
HMMER (v3.3.2)	http://hmmer.org	http://hmmer.org/
Trim_Galore (v0.6.5)	https://www.bioinformatics.babraham.ac.uk/projects/trim_galore/	https://www.bioinformatics.babraham.ac.uk/projects/trim_galore/
aFC (v0.3)	(Mohammadi et al., 2017)^64^	https://github.com/secastel/aFC
ORQAS (Commit 69d5f1a)	(Reixachs-Sole et al. 2020)^21^	https://github.com/comprna/ORQAS
featureCounts (v2.0.6)	(Liao et al., 2014)^65^	https://subread.sourceforge.net
PLINK (v2.0)	(Chang et al. 2015)^66^	https://www.cog-genomics.org/plink/2.0/

**Other**		

DEG/DEI results	This paper	Data S1
Result of translation efficiency in LCL	This paper	Data S2
Result of DIU analysis in LCL	This paper	Data S3
Result of DIU analysis in primary B-cell	This paper	Data S4
Result of DIU analysis for IR-isoforms in primary B-cell	This paper	Data S5
Correlation analysis between IR-isoforms and splicing factors in primary B-cells	This paper	Data S6
Summary statistics of sQTL and colocalized between sQTL and GWAS/COVID-19 data	This paper	Data S7
Summary of read counts at each processing stage for PacBio Iso-Seq datasets	This paper	Table S1
Correspondence table between isoISG and main text isoform name	This paper	Table S2

### Experimental model and subject details

This study was approved by the Ethics Committees of the Medical Research Institute, Tokyo Medical and Dental University.

### Method details

#### Cell culture and RNA extraction

EBV-transformed LCLs (*n* = 94) were obtained from the Medical Research NIGMS Human Genetic Cell Repository at the Coriell Institute, and THP-1 cells were obtained from Japanese Collection of Research Bioresources Cell Bank (JCRB0112.1). Both cells were grown in RPMI 1640 medium with 10% FBS, penicillin (100 unit/mL) and streptomycin (100 μg/mL). They were either unstimulated or stimulated with 50 ng/mL of human IFN-α2 (BioLegend) for 6 h. After centrifugation, cells were lysed in 1 mL of TRIzol (Thermo Fisher Scientific), and high-quality total RNA was extracted from cells using an RNeasy Mini kit (QIAGEN). RNA was treated with DNase using the RNase-Free DNase Set (QIAGEN) during purification. RNA quality and quantity were assessed using a Nanodrop (Thermo Fisher Scientific). The integrity of total RNA was assessed on an Agilent 2100 Bioanalyzer system (Agilent Technologies) using an Agilent RNA6000 Nano kit. The RNA Integrity Number (RIN) criteria for the RNA samples was RIN >9.0. For the single-end RNA-Seq data used for unstimulated samples in the sQTL analysis, total RNA from cultured LCLs was isolated using TRIzol (Thermo Fisher Scientific), treated with DNase, and purified using the RNase-Free DNase Set (QIAGEN) and the RNeasy Mini Kit (QIAGEN). The quality of the RNA was checked using a 2100 Bioanalyzer (Agilent Technologies), and we only used RNA with an RNA Integrity Number (RIN) > 9.0 for RNA-Seq.

#### PacBio RNA-Seq

Full-length cDNA of the LCL samples GM12878 and GM19078 was synthesized from 300 ng of total RNA using NEBNext Single Cell/Low Input cDNA Synthesis & Amplification Module (New England Biolabs). After first strand cDNA synthesis, cDNA was amplified by 12 cycles of PCR. cDNA samples were size selected using a ProNex Size-Selective Purification System (Promega Corporation) as per the PacBio recommendation for standard length cDNA transcripts. Size selected cDNA was used to construct SMRTbell Iso-Seq libraries using SMRTbell Express Template Prep 3.0 (Pacific Biosciences). The Iso-Seq library was run on a Sequel IIe SMRT Cell 8M Tray with a Sequel II Sequencing 2.0 Kit. An extra GM12878 sample was sequenced separately using a Sequel II system with the SMRTbell Express Template Prep 2.0 Kit for comparison.

Raw subreads for each sample were processed into CCS reads as per the manufacturer’s standard pipeline (SMRT Link version 9.0). Only full-length CCS reads that contained primers were extracted and the primers were trimmed using lima version 2.0.0. PolyA tails and artificial concatemers were further removed from the full-length reads using Iso-Seq3 version 3.4.0 with the option *refine --require-polya*. To prevent inconsistencies in the starting and ending positions of the reads and to ensure a unified treatment of transcript variants across different samples, sequences from all samples were merged prior to collapsing. To align the full-length non-chimeric reads to the human genome (hg38), Minimap2 version 2.17^67^ was used with the following settings *--MD -ax splice:hq -uf --secondary=no*. The mapped reads were then collapsed into unique sequences using cDNA-Cupcake version 27.0.0. The collapsed data were validated and annotated by a program (sqanti3_qc program) of SQANTI3 version 5.2.^15^ The annotation was performed with respect to GENCODE v44.^45^ Optional inputs for validation were provided: combined CAGE peak data from refTSS,^47^ TSSclassifier (available in "relaxed" or "strict" modes),^48^ and FANTOM5 CAGE phase 1 and 2,^49^ a polyA motif list and peaks from the PolyASite database,^68^ and short-read splice-junction coverage data. Isoforms across all categories were filtered to include only those not exhibiting intra-priming, defined by having less than 60% adenine downstream of the TTS. Additionally, we exclusively selected isoforms supported by unique short-read junction evidence (≥3 reads) for non-canonical junctions and with a confirmed false reverse transcription switching status, as determined by SQANTI3’s rules filter program. In addition to the SQANTI3 criteria for aggregating junction support across samples, novel non-canonical junctions were required to be supported by ≥ 3 uniquely mapped reads in at least one sample. However, for isoforms classified as FSM to GENCODE annotations, we applied a 'rescue' criterion, allowing the retention of junctions without any supporting reads, to enhance the comprehensiveness of our annotation. To ensure the accuracy of isoform identification under both stimulated and unstimulated conditions, the processing pipeline described above, including junction support analysis with SQANTI3 utilizing condition-specific short-read RNA-Seq data, was uniformly applied to all samples within each condition.

For the quantification of isoform expression from Iso-Seq data, CCS reads were mapped to the human genome using minimap2^45^ with the *-ax splice:hq* parameter. The mapped reads were then quantified using featureCounts,^65^ employing the following parameters: *-L, -t exon, and -g transcript_id*. Subsequently, the raw read counts were normalized to Counts Per Million (CPM) using a custom python script.

#### ONT RNA-Seq

cDNA was synthesized from 100 ng of total RNA with an SMART-seq v4 Ultra Low Input RNA Kit (Takara Bio). SMARTScribe Reverse Transcriptase was used for cDNA synthesis and SeqAmp DNA Polymerase for PCR amplification (12 cycles) of cDNA. After size-selection of cDNA with AMPure XP beads (Beckman Coulter) and quantification of cDNA with a Qubit DNA HS Assay (Thermo Fisher Scientific), 200 fmol of cDNA was sequenced by the Nanopore Ligation Sequencing Kit (SQK-LSK110; ONT) with a MinION Flow Cell (R9.4.1, FLO-MIN106; ONT) for 72 h. Basecalling was performed using Guppy v4.4.1 with the SUP (super high accuracy) mode (Wick et al., 2019). The four LCL samples (GM18943, GM19066, GM19075, GM19078) were used for sequencing. For THP1 cells, the Nanopore Ligation Sequencing Kit (SQK-LSK114; ONT) with a PromethION Flow Cell (R10.4.1, FLO-MIN114; ONT) was used, adopting a similar sequencing and basecalling methodology. For THP1 cells, 6 samples (three replicates each, with and without stimulation) were multiplexed using custom sequences that combined the barcode and ISPCR primer to tag the PCR products.

Reads from raw data were pre-processed with Pychopper v2 (ONT) to extract full-length reads based on the presence of adapters and poly-A tails. The FL reads were further processed using the FLAIR pipeline.^50^ The FL reads were mapped to the human genome using the flair-align module with default parameters. In the splice junction correction step using the flair-correct module, we used splice-junction coverage data of LCL short-read RNA-Seq data (*n* = 114, unstimulated = 20 and stimulated = 94). After a further collapsing step using the flair-collapse module, we extracted isoforms whose 5′-ends were located within 100 bp from the TSS annotated by refTSS^48^ and with a minimum number of three supporting reads with MAPQ ≥1. This threshold follows Tang et al.,^50^ where benchmarking showed that these criteria provided accurate quantification and balanced sensitivity and precision. We validated and annotated the generated gtf files using SQANTI3.

For the quantification of isoform expression, reads were mapped to the isoISG reference sequences using minimap2 with the -ax map-ont option. Mapped reads were then quantified using Salmon^51^ with the following parameters: *--ont and --noErrorModel*. Only read length >500bp was used for the quantification.

#### Short read RNA-Seq

Poly-A tailed mRNAs were selected from 1 μg of total RNA from LCLs using a NEBNext Poly(A) mRNA Magnetic Isolation Module (New England Biolabs). cDNA libraries were prepared from 10 ng of poly-A tailed RNA using NEBNext UltraDirectional RNA Library Prep Kit for Illumina (New England Biolabs), and pair-end sequencing (150 bp) of libraries was performed with a NovaSeq 6000 (Illumina). For the single-end RNA-Seq data used for unstimulated samples in the sQTL analysis, total RNA from cultured LCLs was additionally subjected to rRNA reduction using the Ribo-Zero rRNA Removal Kit (Illumina). The RNA-Seq library was constructed using TruSeq RNA Sample Prep Kits v2 (Illumina). Sequencing was performed using the Illumina HiSeq 2000 sequencer (101 bp reads).

For primary B-cells, RNA-Seq data were downloaded from the GEO database (GSE118875).^24^ We obtained 12 samples from healthy individuals (４ each of IFN-I-stimulated [24 h], and [72 h], and unstimulated [0 h] conditions). The total read numbers were important for DIU analysis because they were used to calculate the isoform fraction. We assessed the read numbers of all samples and removed one unstimulated sample due to low read number (about half the number of the other unstimulated samples).

Adapters and low-quality tails were trimmed from reads using fastp version 0.21.0^63^ with the following parameters: *-q 20*, *-l 30*. The quantification of isoISG transcripts was performed using Salmon^51^ with parameters: *--validateMappings*, *--seqBias*, *--gcBias and --minScoreFraction 0.8*. These parameters were applied uniformly to all short-read RNA-Seq data, including both single- and paired-end reads. For analysis of differential expression, we used the R package tiximport^69^ to generate an isoform/gene count matrix, filtered >0.1 TPM at least 10% of samples. Differential expression analyses were conducted using the R package DESeq2 v１.３４^70^ for both genes and isoforms. The list of samples used in the DEG analysis and results of DEG and DEI analysis are provided in Data S1.

#### Comparison of correlations across sequencing technologies

Isoform expression matrices from ONT, PacBio, and Illumina technologies were analyzed using R. Isoforms with zero expression in all samples were excluded to focus the analysis on expressed isoforms. Correlation of isoform expression levels was then computed across all samples using the Pearson method with the cor.test function in R. Median r^2^ values were calculated for each sequencing technology. To simulate RNA-Seq datasets that accurately reflect actual isoform counts, we utilized Polyester.^59^ This simulation involved generating reads for each isoform in quantities that match those mapped to each isoform in the experimentally obtained RNA-Seq data, ensuring the source of the reads was unequivocal. Specifically, Polyester was used to generate simulated short-read datasets with the following parameters: *error_model="uniform", reads_per_transcript=expression_levels, fold_changes=fold_changes, paired=TRUE, num_reps=c(1), readlen=150*. We used the error rate of 0.00109 for NovaSeq 6000.^71^ Quantification of simulated data was done using Salmon, with parameters aligned to the original RNA-Seq data. Correlation analysis of this data followed the procedure outlined above.

For assessing the concordance of splice junctions detected by short-read sequencing with those identified in long-read sequencing data, junction files generated by STAR^57^ (for short-read data) and SQANTI3 (for long-read data) were utilized. A custom Python script was used to calculate the concordance rate between the isoISG annotation and the splice junctions detected in short-read data, specifically considering junctions supported by three or more unique reads in each sample.

#### Luciferase assay

The CDSs of *IRF3* were cloned into the pcDNA3.1D/V5-His-TOPO vector (Thermo Fisher Scientific) using cDNAs from LCL. The 5′-UTR sequences of *IRF3* were cloned into the pFL-SV40 vector (Addgene) just upstream of the CDS of luciferase gene. We cultured HEK-293 cells in DMEM supplemented with 10% fetal bovine serum. For the CDS assay, each *IRF3* CDS vector was transfected in combination with pEF-BOS-hTLR3-FLAG vector (provided by Dr. Matsumoto, Hokkaido University), p55C1B-Luc vector (provided by Dr. Fujita, Kyoto University), and the pGL4.74 vector (hRluc/TK as an internal control) (Promega) into cells using the X-tremeGENE HP Transfection Reagent (Roche). After 24 h of incubation, the cells were stimulated with 10 μg poly (I:C) (Enzo Life Sciences) for an additional 24 h. We then collected the cells and measured luciferase activity using the Dual-Luciferase Reporter Assay (Promega). For the 5′-UTR assay, each of *IRF3* 5′-UTR vector (comprising both 5′-UTR sequence of *IRF3* and a luciferase CDS) was used instead of the *IRF3* CDS vector and p55C1B-Luc vector.

#### Translational efficiency

We estimated translational efficiency at the isoform level as previously described.^72^ The ribo-seq data (GSE61742)^22^ and RNA-Seq data from the GEUVADIS project^46^ were used, selecting samples from 52 common Yoruba individuals across the two expression datasets. After trimming with Trim_Galore, the reads were processed with ORQAS^21^ to calculate translational efficiency. Default parameters were used for the calculations. A positive DEI was defined as log2FC > 0.5. The translational efficiency for isoISGs is provided in Data S2.

#### Isoform switching analysis

To facilitate the analysis of isoform switching, isoform abundance was first quantified separately for LCL and primary B-cells by aligning short-read data against the isoISG sequences identified from long-read sequencing. This alignment was performed using Salmon, which provides precise expression quantification. This prepared dataset was used to detect isoform switching events between unstimulated and stimulated samples using a DEXSeq-based test implemented in IsoformSwitchAnalyzeR v1.8.0.^18^ Significant isoform switching was defined as events with a difference in dIF >0.05 with FDR <0.05. We used the expression matrix of isoISG, which was quantified by Salmon, with a gene expression cutoff >0.1 (TPM). The results are provided in Datas S3 and S4, respectively. AS events were evaluated in the increased and decreased isoforms and the consequences underlying the isoform switching were evaluated using the analyzeSwitchConsequences function in IsoformSwitchAnalzeR. For the detection of consequences such as coding potential, protein domain, signal peptide, and intrinsically disordered regions, we used HMMER v3.3.2, CPAT,^54^ SignalIP-5.0,^53^ and IUPred3,^55^ respectively. The enrichment analysis of gain of AS in isoform switching was performed using the extractSplicingEnrichment function.

#### Detection of domain loss in spliced isoforms

The relationship between AS events and the loss of functional domains in isoISG isoforms was investigated. First, the pfam domain^56^ in the isoform sequence was predicted by HMMER hmmscan, and the presence or absence of the domains was compared between isoforms within each gene and isoforms with missing domains were identified using bedtools.^62^ Next, the association between AS events and the loss of domains in each isoform was determined by examining the overlap between the genomic coordinates at which the AS occurred and the location of the lost domains. The genomic coordinates of AS events predicted by IsoformSwitchAnalyzeR were used in this analysis; a chi-square test was used to determine if there was a significant difference in the number of domains lost due to each AS event before and after IFN-I stimulation.

For the isoforms that lost domains due to IR events, we further performed differential IR isoform usage analyses. Specifically, the expression of isoISG isoforms quantified by Salmon were aggregated for IR and non-IR isoforms, and their fractions in each gene were calculated. The R package DRIMSeq v1.14.0^60^ was used to identify differential IR isoform usage with the following parameters: *min_samps_feature_prop=0.01*, *min_samps_gene_expr=3*, *min_gene_expr=10*. The The results of differential IR isoform usage analyses are provided in Data S5.

#### Correlation analysis with splicing factors

The read counts of short-read RNA-Seq data were normalized using the variance-stabilizing transformation (vst) method in DESeq2. The Spearman correlation between the expression levels of the IR-isoforms of ISGs and splicing factors was calculated using the cor function of the R package WGCNA.^73^ The list of ISGs and splicing factors were obtained from the Molecular Signatures Database (MSigDB) v7.5.1. Splicing factors containing IR-isoforms were excluded from the analysis. The results are provided in Data S6.

#### Prediction of RBP binding sites

To investigate the association between SR family genes and IR isoforms, we searched for binding motifs of SR family genes in regions where IR occurs or does not occur (exons and introns) using RBPmap.^52^ For the prediction of RBP binding sites, we used binding motifs of the SRSF genes (*SRSF5*, *6*, and *8*) preinstalled in RBPmap and *SRSF6*.^74^ The predicted binding sites were counted for exons and introns, each divided into 10 bins and then aggregated by regions where IR occurs (IR exons and introns) and regions where IR does not occur (non-IR exons and introns) for each gene. The predicted numbers were compared to determine if there was a significant difference in regions where IR was observed or not observed. Wilcoxon rank-sum test (one-sided) was performed in each bin between regions with and without IR events, respectively, and all comparisons were significant (FDR <0.001).

#### QTL analysis

We used short-read RNA-Seq data of IFN-I-stimulated and unstimulated B-cell lines (LCL) from a Japanese population (*n* = 94) to perform the QTL analysis. Stimulated data were sequenced as described above. Unstimulated data were obtained from another project (unpublished) and were single-end sequenced. Both the stimulated and unstimulated samples for the sQTL analysis came from the same donors. Genetic variant (SNPs) data were downloaded in variant calling format (vcf) from the 1KG project (http://ftp.1000genomes.ebi.ac.uk/), and variants with a minor allele frequency (MAF) greater than 0.05 were selected using Plink^66^ software.

We performed junction-based sQTL analysis using LeafCutter v0.2.9.^36^ First, short-read RNA-Seq data were mapped to the human genome using STAR v2.7.3a with parameters: *--twopassMode Basic --outSAMstrandField intronMotif*, and then junctions were extracted from mapped reads using helper scripts provided by leafcutter. The junction reads were further clustered and counted using the leafcutter_cluster.py program with the parameters: *-l 500000*, *-m 50*, and intron excision ratios were calculated. We used junctions that were used in >10% of samples. Normalization of the excision ratios was performed with PEER normalization using 15 hidden factors to eliminate batch effects between experiments.^58^ We also conducted isoform ratio QTL analysis. Gene expression in isoISG was quantified with Salmon and normalized with the TMM-method using edgeR v3.36.0.^75^ Genes with TPM >1 in 10% of samples were further normalized with PEER using 15 hidden factors. We performed the eQTL analysis in permutation pass mode using QTLtools v1.3.1^61^ with the parameters: *--permute 1000* and *–window 1000,000* and selected associations to the lead SNP per gene. The effect size for sQTL was calculated using allelic Fold Change (aFC) with parameters: *--log_xform 1*, *--log_base 2*.^64^

We evaluated the co-localization of sQTL effects and GWAS by RTC analysis^37^ in RTC mode of QTLtools with the parameter *--normal*. GWAS summary data (all associations v1.0.2) were downloaded from the EMBL-EBI GWAS catalog. The summary data of COVID-19 GWAS meta-analyses (A2_ALL_leace_23andme, very severe respiratory confirmed COVID vs. population) were obtained from COVID-19 HGI release7. Results of the colocalization analyses and samples are provided in Data S7.

#### Image generation and visualization

The images were generated using the R package and refined for visual clarity. Subsequently, the plots were further enhanced and modified using graphic design software, Adobe Illustrator 2020, to ensure optimal data representation. The correspondence between isoform IDs shown in the figures and isoISG IDs is represented in Table S2.

## Supplemental information

Document S1. Figures S1–S6 and Tables S1 and S2

Data S1. DEG/DEI results, related to Figures 2 and 3 Data S1a. DEG (gene level) of LCL. DataS1b. DEI (transcript level) of LCL. Data S1c. DEG (gene level) of primary B-cell. DataS1d. DEI (transcript level) of primary B-cell [0h vs. 24h]. DataS1e. DEI (transcript level) of primary B-cell [0h vs. 72h]. DataS1f. Lists of samples used in DEG/DEI analysis.

Data S2. Result of translation efficiency in LCL, related to Figure 2 (Columns) gene: The gene name, isolSG: Isoform identifier of isoISG annotation, TE: Translation efficiency, Periodicity: Measure of how often reads occur at the annotated frame compared to alternative frames, Uniformity: Proportion of maximum entropy, indicating the distribution uniformity of reads along the ORF.

Data S3. Result of DIU analysis in LCL, related to Figure 2 DataS3a. DIU analysis of LCL (|dIF| > 0.05). DataS3b. Enriched AS event analysis of LCL (|dIF| > 0.05). DataS3c. Functional consequence analysis of LCL (|dIF| > 0.05).

Data S4. Result of DIU analysis in primary B cells, related to Figure 3 DataS4a. DIU analysis of Primary B-cells (dIF >0.05). (Columns) isoform_id: Identifier for the isoform, condition_1: Control condition, condition_2: IFNa2 condition, dIF: Difference in isoform fraction between conditions (IF2 - IF1), pvalue: P-value for the differential isoform usage, padj: Adjusted p-value for multiple testing corrections, IF1: Isoform fraction in Control condition, IF2: Isoform fraction in IFNa2 condition, gene_name: Name of the gene associated with the isoform. DataS4b. Enriched AS event analysis of primary B-cell (|dIF| > 0.05). (Columns) condition_1: Unstimulated condition, condition_2: Stimulated condition, AStype: Type of AS event, nUp: Number of genes with a gain of the splicing type, nDown: Number of genes with a loss of the splicing type, propUp: Proportion of genes with a gain of the splicing type, propUpCiLo: Lower confidence interval of propUp, propUpCiHi: Upper confidence interval of propUp, propUpPval: P-value for propUp, propUpQval: Adjusted p-value for propUp, Significant: Significance indicator. DataS4c. Functional consequence analysis of primary B-cell (|dIF| > 0.05). (Columns) condition_1: Unstimulated condition, condition_2: Stimulated condition, AStype: Type of AS event, conseqPair: Set of opposite consequences, feature: Description of the consequence (with the opposite in parentheses), propOfRelevantEvents: Proportion of genes with the consequence, propCiLo: Lower confidence interval of propOfRelevantEvent, propCiHi: Upper confidence interval of propOfRelevantEvents, propPval: P-value for propOfRelevantEvents, nUp: Number of genes with the consequence, nDown: Number of genes with the opposite consequence, propQval: Adjusted p-value for propOfRelevantEvents.

Data S5. Result of DIU analysis for IR isoforms in primary B cells, related to Figure 5 Data S5a. Results of differential IR isoform usage analyses [0h vs. 24h]. Data S5b. Results of differential IR isoform usage analyses [0h vs. 72h]. Data S5c. Isoform fraction (IF) values of IR isoforms.

Data S6. Correlation analysis between IR isoforms and splicing factors in primary B cells, related to Figure 5

Data S7. Summary of sQTL and ir-QTL analyses, including colocalization with GWAS (including COVID-19 Host Genetics Initiative) data, related to Figure 6 Data S7a. sQTL (leafcutter) results. Data S7b. ir-QTL results. Data S7c. Colocalization of GWAS and sQTL (leafcutter). Data S7d. Colocalization of GWAS and ir-QTL. Data S7e. Colocalization of COVID-19 and ir-QTL.

Document S2. Article plus supplemental information
