# Supplementary material for: Functional and dynamic profiling of transcript isoforms reveals essential roles of alternative splicing in interferon response
Source: Cell Genom. 2024 Sep 16;4(10):100654. doi: 10.1016/j.xgen.2024.100654 (PMC11602592; doi:10.1016/j.xgen.2024.100654)
Supplement: Document S1. Figures S1–S6 and Tables S1 and S2 [file mmc1.pdf]

Cell Genomics, Volume *4*

Supplemental information

Functional and dynamic profiling of transcript isoforms reveals essential roles of alternative splicing in interferon response

Mahoko Takahashi Ueda, Jun Inamo, Fuyuki Miya, Mihoko Shimada, Kensuke Yamaguchi, and Yuta Kochi

Supplemental Information File - Table of Contents

1. Supplemental Figures

Figure S1. Validation of isoISG annotation and comparative analysis of sequencing technologies in LCL, related to Figure 1.

Figure S2. AS and its functional consequences via isoform switch during the initial phase of IFN-I response, related to Figure 2.

Figure S3. Validation of isoISG annotation detected in primary B-cell, related to Figure 4.

Figure S4. Isoform switches in primary B-cell during the late phase of IFN-I response, related to Figure 5.

Figure S5. AS and its functional consequences via Isoform switch across cell types and IFN stimulations beyond IFNa2, related to Figure 2.

Figure S6. sQTL analysis workflow, related to Figure 6.

2. Supplemental Tables

Table S1. Summary of read counts at each processing stage for PacBio Iso-Seq datasets, related to Figure 1.

Table S2. Correspondence table between isoISG annotation and main figure isoform name, related to STAR Methods.

Supplemental Figures

Supplementary Fig. 1

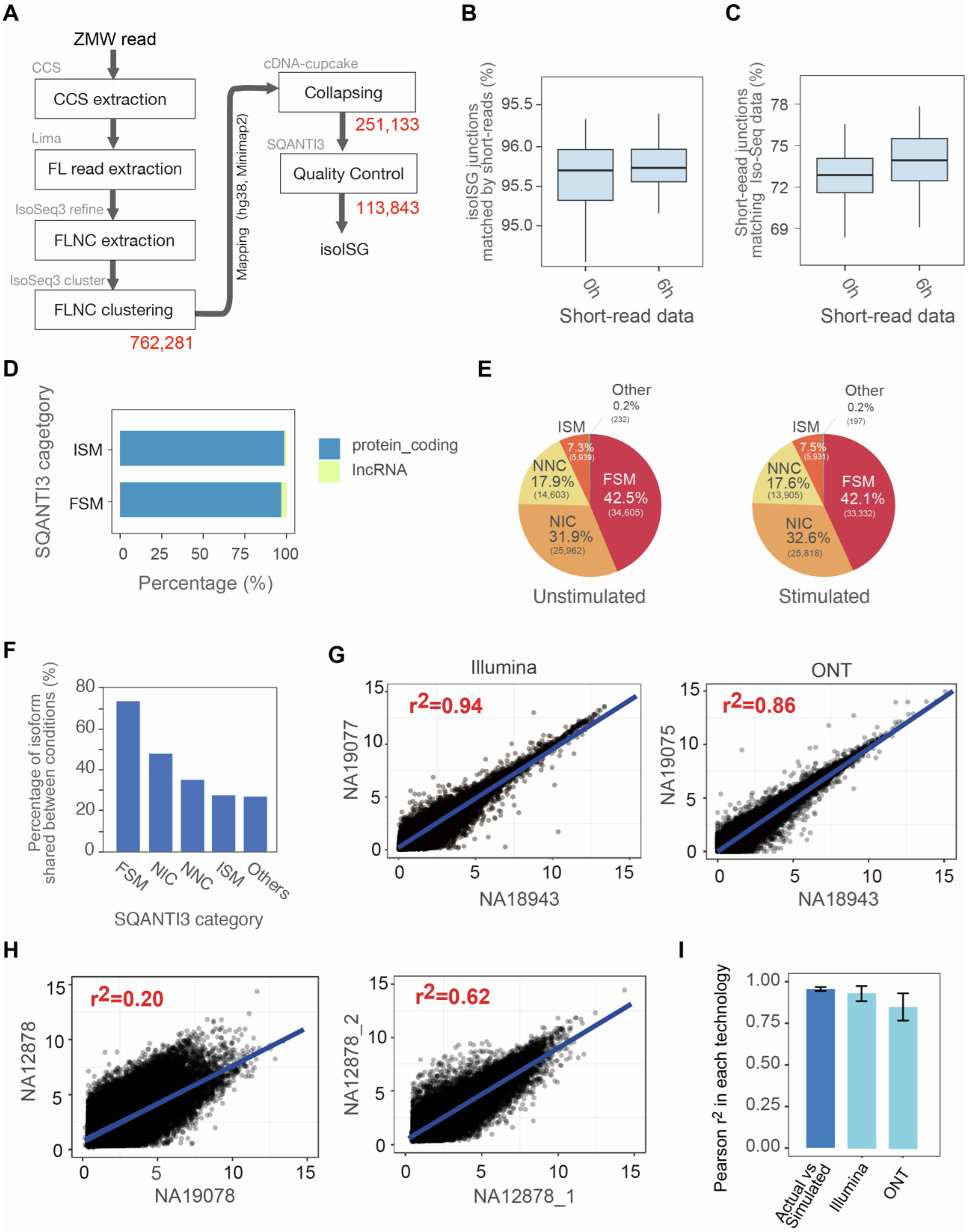

Figure S1. Validation of isoISG annotation and comparative analysis of sequencing technologies in LCL, related to Figure 1

(**A**) Data processing steps of PacBio Iso-Seq sequencing raw output. The tool used in each step is shown in gray, and the number of combined reads after collapsing in red. Read numbers for each sample before the collapsing step are shown in Table S1. ZMW: zero-mode waveguides, CCS: Circular consensus sequencing, FL: full-length, FLNC: full-length non-concatemer.

(**B**) Boxplot shows the percentage of splice junctions from all isoforms in isoISG that are covered by short-read sequencing data (either 0h or 6h). 20 short-read data were analyzed for each condition, and error bars indicate 95% confidence intervals.

(**C**) Boxplot shows the percentage of splice junctions identified in short-read sequencing data that are also found in Iso-Seq data, comparing within the same experimental condition (either 0h or 6h). 20 short-read data were analyzed for each condition, and error bars indicate 95% confidence intervals. Only junctions supported by at least 3 reads were used.

(**D**) Bar chart illustrates the Ensembl/GENCODE biotype composition of isoforms classified into the FSM and ISM categories as analyzed by SQANTI3. It shows the percentage breakdown of isoforms into two main biotypes: protein coding (blue) and lncRNA (light green). Only biotypes representing at least 1% of the total are displayed.

(**E**) SQANTI3 categories of unstimulated (left) and IFN-α2-stimulated (right) conditions. Each segment denotes the proportion and the number of isoforms categorized as Full Splice Match (FSM), Novel In Catalog (NIC), Novel Not in Catalog (NNC), and Other. Control: Un-stimulated samples.

(**F**) Graph displays the percentage of isoforms in the isoISG annotation that are shared between unstimulated and stimulated conditions within each structural category.

(**G**) Representative scatter plots of isoform expression levels from Illumina (left) and ONT (right) technologies. Each point represents an isoform, with the blue line of best fit indicating the correlation.

(**H**) Representative scatter plots of isoform expression levels from PacBio between inter-samples (left) and intra-samples (right). Each point represents an isoform, with the blue line of best fit indicating the correlation.

(**I**) Bar chart illustrating Pearson r^2 values for isoform expression correlation. The first bar indicates average correlation between actual and Polyester-simulated Illumina datasets. The subsequent bars represent average intra-technology correlations for Illumina and ONT, with blue denoting intra-sample and light blue for inter-sample comparisons. Error bars depict the standard error of the mean.

Supplementary Fig. 2

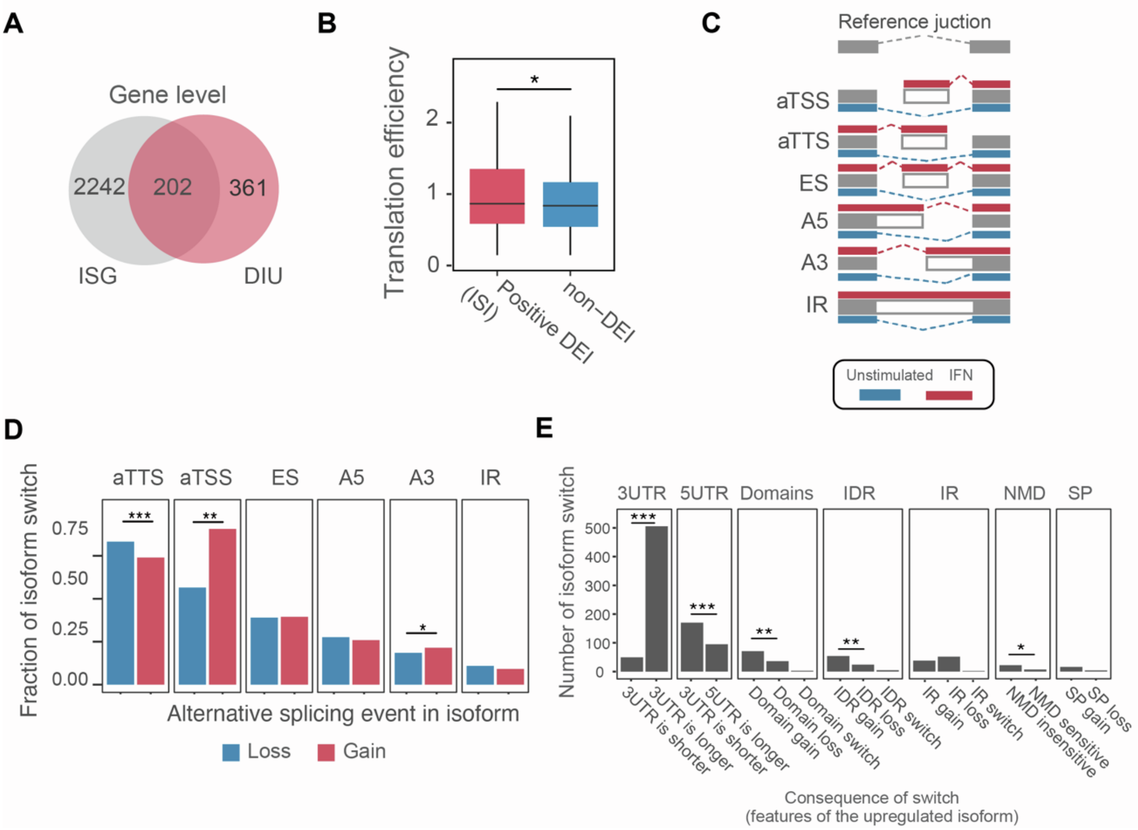

Figure S2. AS and its functional consequences via isoform switch during the initial phase of IFN-I response, related to Figure 2

(**A**) Overlap of DIU and ISG at gene level.

(**B**) Translation efficiency of DEI groups. *, *FDR* < 0.05 (Wilcoxon rank sum test).

(**C**) Six types of AS events defined in IsoformSwitchAnalyzeR. aTSS; alternative transcription start site, aTTS; alternative termination site, ES; exon skipping, A5; alternative 5'end donor site, A3; alternative 3'end acceptor site, IR; intron retention. The AS event of each isoform is defined by comparing the junctions with those of a hypothetical mRNA that is constructed by concatenating all exons of known isoforms within the same gene.

(**D**) Fraction of isoform switches associated with AS events in primary B-cells induced by IFN-I stimulation. Red bars represent gains and blue bars represent losses in the

proportion of each AS event type. *, FDR < 0.05; **, *FDR* < 0.01; ***, *FDR* < 0.001 (chi-squared test).

(**E**) Number of isoform switches and associated functional consequences 6 hours post-IFN-I stimulation. Bars indicate the number of upregulated isoforms with changes in each functional category. *, *FDR* < 0.05; **, *FDR* < 0.01; ***, *FDR* < 0.001.

Supplementary Fig. 3

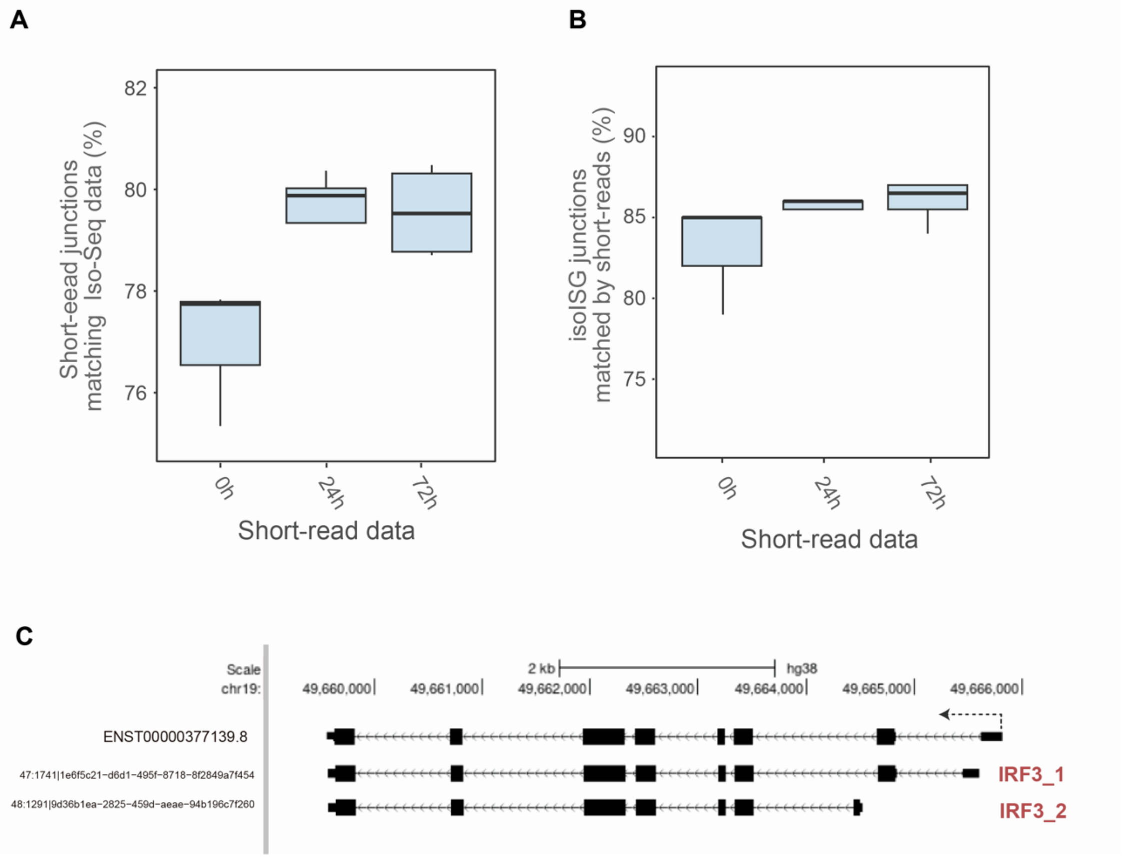

Figure S3. Validation of isoISG annotation detected in primary B-cell, related to Figure 4

(**A**) Boxplot shows the percentage of splice junctions identified in short-read sequencing data that are also found in Iso-Seq data, comparing within the two conditions (either unstimulated or stimulated). 3, 4, and 4 samples of short-read RNA-Seq data were analyzed for each condition of 0h, 24h, and 72h, respectively. Error bars indicate 95% confidence intervals.

(**B**) Boxplot shows the percentage of splice junctions from all isoforms in isoISG covered by short-read sequencing data at 0h, 24h, and 72h post-stimulation, analyzing 3, 4, and 4 short-read RNA-Seq samples for each respective condition. Error bars indicate 95% confidence intervals.

(**C**) Schematic representation of novel IRF3 isoforms in ONT RNA-Seq. These annotations were generated in LCL by FLAIR pipeline. The reference isoform (ENST00000377139.8) is shown for comparison.

Supplementary Fig. 4

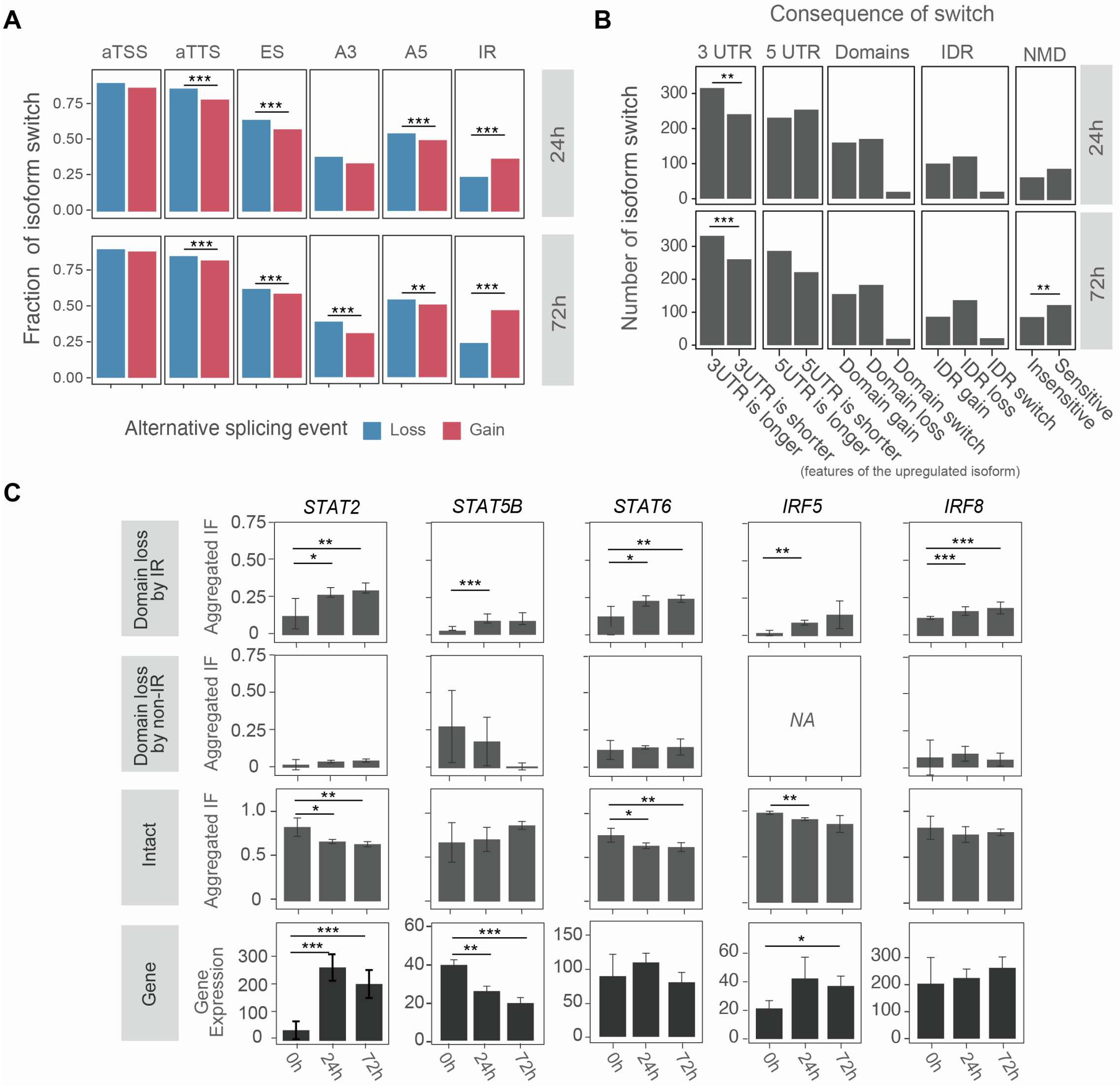

Figure S4. Isoform switches in primary B-cell during the late phase of IFN-I response, related to Figure 5

(**A**) Fraction of isoform switches associated with AS events in primary B-cells induced by IFN-I stimulation (24 h and 72 h). Red bars represent gains and blue bars represent losses in the proportion of each AS event type. *, FDR < 0.05; **, *FDR* < 0.01; ***, *FDR* < 0.001 (chi-squared test).

(**B**) Number of isoform switches and associated functional consequences 24 and 72 hours post-IFN-I stimulation. Bars indicate the number of upregulated isoforms with changes in each functional category. *, *FDR* < 0.05; ***, *FDR* < 0.001.

(**C**) Aggregated isoform fractions of domain-lost isoforms caused by IR or other mechanisms, isoforms retaining intact functional domains, and the total gene-level expression (measured by TPM) for *STAT2*, *STAT5B*, *STAT6*, *IRF5*, and *IRF8*. These metrics are displayed for unstimulated and IFN-I-stimulated samples (24 and 72 hours). Statistical significance of the differences in the aggregated isoform fraction and gene expression between the stimulated and unstimulated conditions is denoted by asterisks as determined by the Mann-Whitney two-tailed test: *, FDR < 0.05; **, FDR < 0.01; ***, FDR < 0.001. Error bars represent 95% confidence intervals. 'N/A' indicates that data are not available or not applicable.

Supplementary Fig. 5

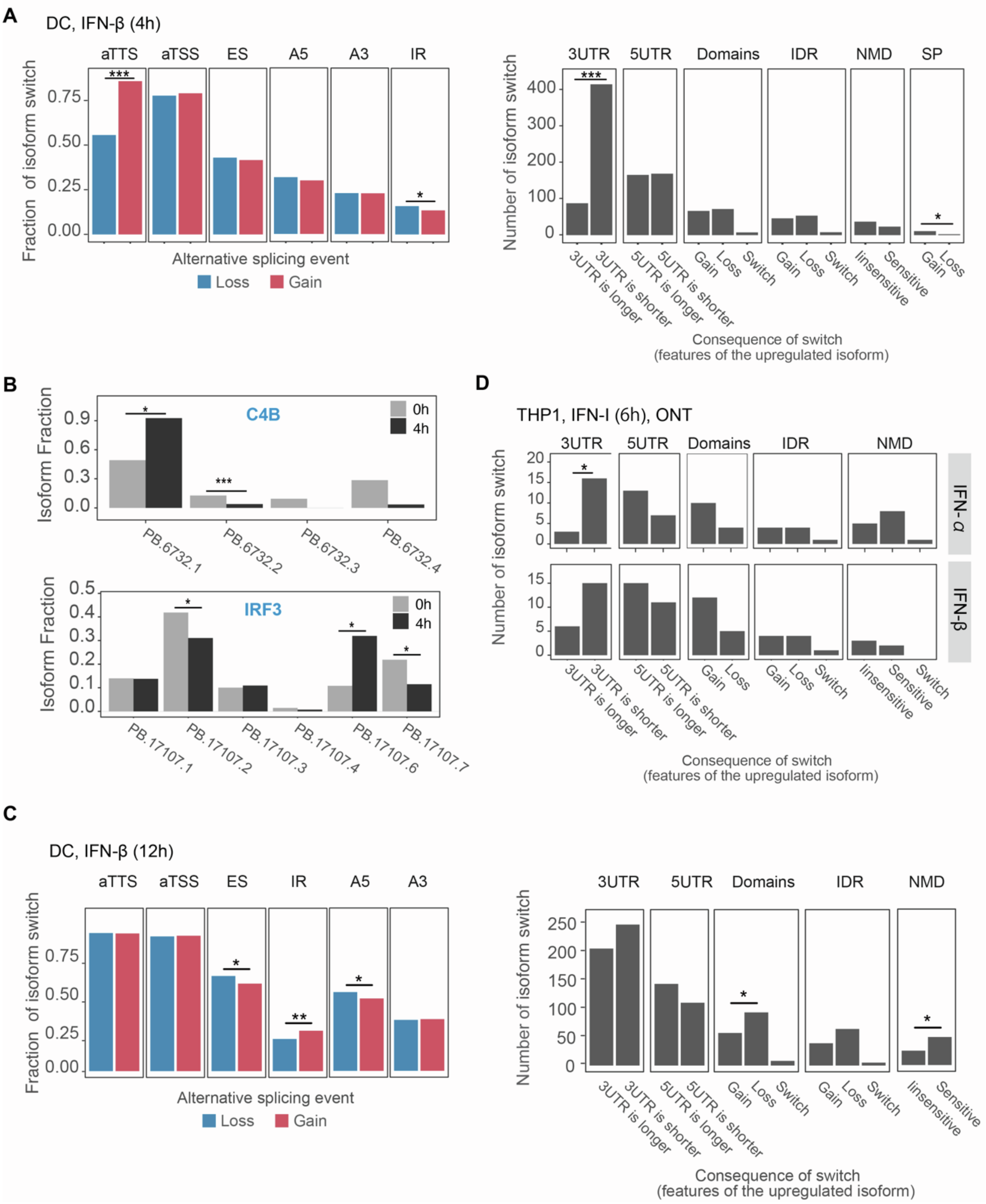

Figure S5. AS and its functional consequences via isoform switch across cell types and IFN stimulations beyond IFNa2, related to Figure 2

(**A**) Left: Fractions of isoform switches associated with AS events in DCs 4 hours after IFN-β stimulations. Red bars represent gains and blue bars represent losses in the proportion of each AS event type. Right: Number of isoform switches and associated

functional consequences in DCs 4 hours after IFN-I stimulation. Bars indicate the number of upregulated isoforms with changes in each functional category.

(**B**) Representative examples of functional consequences via isoform switch after 4 hours of IFN-α and IFN-β stimulation. Top: Isoform switch in *C4B*. Error bars, 95% confidence intervals. Bottom: Isoform switch in *IRF3*. *, FDR < 0.05 (Mann–Whitney two-sided test). Error bars, 95% confidence intervals.

(**C**) Left: Fractions of isoform switches associated with AS events in DCs 12 hours after IFN-β stimulations. Red bars represent gains and blue bars represent losses in the proportion of each AS event type. Right: Number of isoform switches and associated functional consequences in DCs 12 hours after IFN-I stimulation. Bars indicate the number of upregulated isoforms with changes in each functional category.

(**D**) Enrichment of functional consequences associated with isoform switches in THP1 cells using ONT RNA-Seq data, comparing conditions at 0 hours and 6 hours post-stimulation. The number of upregulated isoforms with changes in each functional category is shown.

Keys in panels A, C, and D: aTSS; alternative transcription start site, aTTS; alternative termination site, ES; exon skipping, A5; alternative 5' splice site, A3; alternative 3' splice site, IR; intron retention, IDR; Intrinsically disordered regions, SP; signal peptides, and NMD; nonsense-mediated mRNA decay. All panels share the statistical significance: *FDR < 0.05; **FDR < 0.01; ***FDR < 0.001 (chi-squared test).

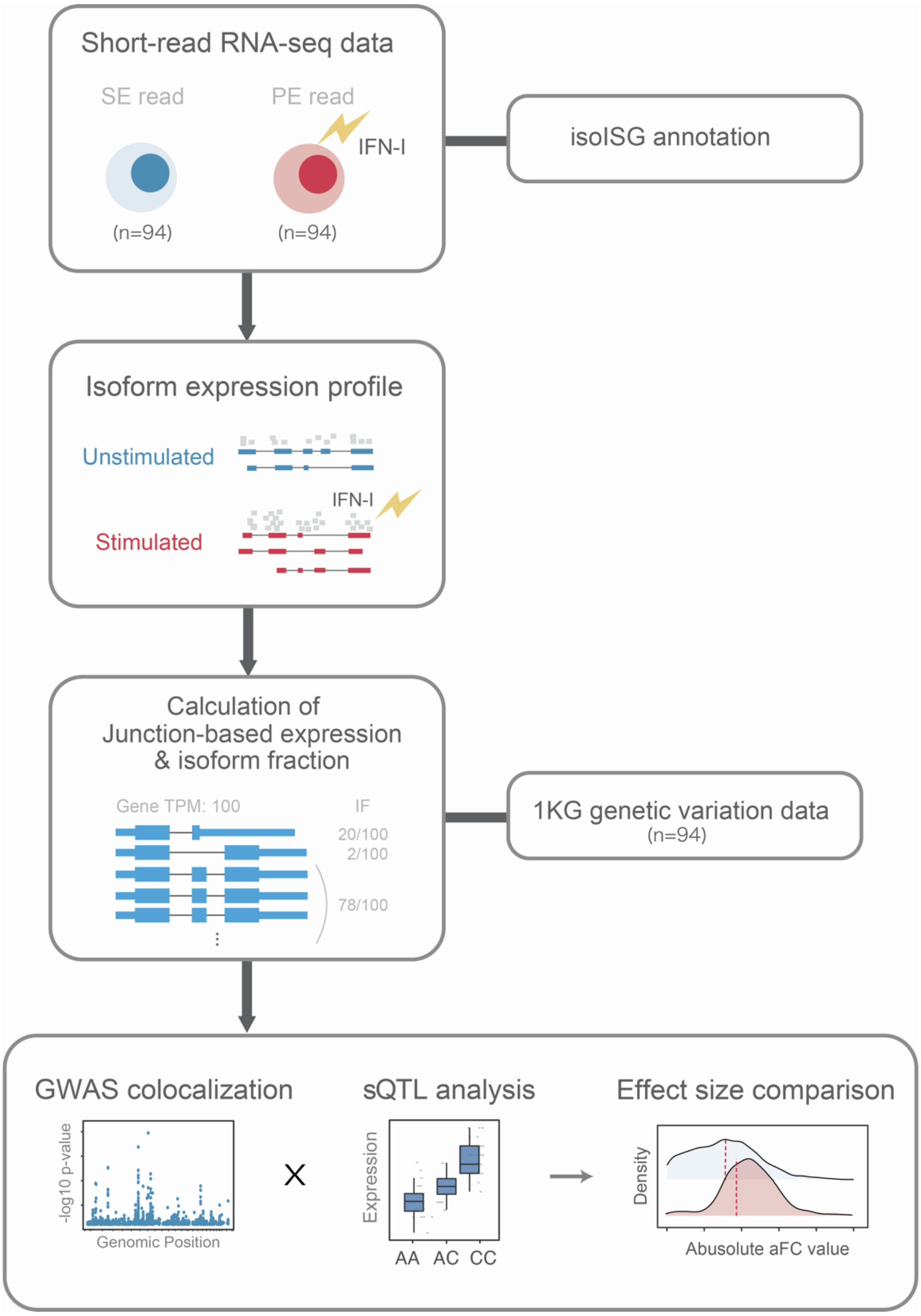

Figure S6. sQTL analysis workflow, related to Figure 6

Schematic workflow of the sQTL analysis. Short-read RNA-Seq data (SE reads for unstimulated samples and PE reads for IFN-I stimulated samples at 6 hours, n=94 each) are used to generate isoform expression profiles with isoISG annotation. These profiles,

along with 1KG genetic variation data, are utilized to calculate junction-based expression and isoform fractions for sQTL analysis. Downstream analyses include effect size comparison of sQTL and sQTL-GWAS colocalization. SE: short-read and PE: paired-end. Note that 20 of the 94 PE samples, specifically from the IFN-I stimulated condition, were also used in DEG analysis (see Figure 1A). All RNA-Seq samples (SE and PE) and genetic variation data are from the same donors.

Supplemental Tables

Table S1. Summary of read counts at each processing stage for PacBio Iso-Seq datasets, related to Figure 1

This table summarizes the read counts at each processing stage for the PacBio Iso-Seq datasets. Each color corresponds to a specific processing step (tool name). The "Label" column indicates the stimulation condition, and the percentages in parentheses represent the proportion of reads remaining from the previous step.

Table S1A. Initial step read counts (quality filtering)

			CCS		Lima
**Sample**	**IFN**	**Label**	**ZMW input**	**Pass**	**#Full-length**
NA12878	6h	NA12878_6h_1	4,152,420	2,587,578 (62.31%)	2,331,325 (90%)
NA12878	0h	NA12878_0h	7,170,755	3,993,039 (55.69%)	3,645,262 (91%)
	6h	NA12878_6h_2	6,660,872	3,474,937 (52.17%)	3,178,440 (91%)
NA19078	0h	NA19078_0h	7,137,711	3,555,018 (49.81%)	3,188,059 (90%)
	6h	NA19078_6h	6,622,303	3,352,416 (50.62%)	3,645,262 (91%)

Table S1B. Later step read counts (refinement, clustering, and collapsing).

			Isoseq3 refine		Isoseq3 cluster	cDNA-cupcake collapse
**Sample**	**IFN**	**Label**	**#Read_flnc**	**#Read_flnc _polya**	**#Read_ clusterd**	**#Uniq_ transcript**
NA12878	6h	NA12878_6h_1	2,314,564	2,313,166	123,698	92,478
NA12878	0h	NA12878_0h	3,640,780	3,637,458	163,081	104,672
	6h	NA12878_6h_2	3,175,135	3,172,197	143,120	94,099
NA19078	0h	NA19078_0h	3,183,326	3,180,190	165,811	106,799
	6h	NA19078_6h	3,640,780	3,039,074	166,571	106,979

Note:

Sample: Sample identifier.
IFN: Interferon alpha 2 stimulation condition (0h for unstimulated, 6h for stimulated for 6 hours).
Label: Sample label including stimulation condition.

ZMW input: Number of Zero-Mode Waveguides (ZMW) input reads.
Pass: Number of reads passing the quality filter, with the percentage of ZMW input reads in parentheses.
#Full-length: Number of reads with primers at both ends, indicating full-length reads, with the percentage of passing reads in parentheses.
#Read_flnc: Number of full-length non-chimeric (FLNC) reads.
#Read_flnc_polya: Number of polyadenylated FLNC reads.
#Read_clustered: Number of reads after clustering.
#Uniq_transcript: Number of unique collapsed transcript sequences.

Abbreviations:
CCS: Circular Consensus Sequencing, ZMW: Zero-Mode Waveguides, FLNC: Full-Length Non-Chimeric

Table S2. Correspondence table between isoISG annotation and main figure isoform name, related to STAR Methods

This table provides the correspondence between isoform IDs used in the main figures and their corresponding isoISG annotation IDs.

Figure	name in figure	Isoform name
Fig.2E	C4B_1	PB.6732.2
	C4B_2	PB.6732.2
	C4B_3	PB.6732.4
Fig.3D	MAP2K5_1	PB.14209.1
Fig. 4A	IRF3_1	PB.17107.4
	IRF3_2	PB.17107.6
Fig.5D	STAT1_1	PB.2923.19
	STAT1_2	PB.2923.172
	STAT1_3	PB.2923.173
	STAT1_4	PB.2923.85
	STAT1_5	PB.2923.381
	STAT1_6	PB.2923.278
	STAT1_7	PB.2923.182
	STAT1_8	PB.2923.91
	STAT1_9	PB.2923.179
	STAT1_10	PB.2923.148
	STAT1_11	PB.2923.178
	STAT1_12	PB.2923.216
	STAT1_13	PB.2923.63
	STAT1_14	PB.2923.280
	STAT1_15	PB.2923.277
	STAT1_16	PB.2923.180
Fig.6D	IRF5-1	PB.8362.81
Fig.6E	STAT6_1	PB.12301.6
Fig.6G	TYK2_1	PB.16552.49
